# BayesMetab: treatment of missing values in metabolomic studies using a Bayesian modeling approach

**DOI:** 10.1186/s12859-019-3250-2

**Published:** 2019-12-20

**Authors:** Jasmit Shah, Guy N. Brock, Jeremy Gaskins

**Affiliations:** 1 Department of Population Health, The Aga Khan University, Nairobi, Kenya; 2 Department of Biomedical Informatics, The Ohio State University, Columbus, OH 43210 USA; 3 Department of Bioinformatics and Biostatistics, University of Louisville, Louisville, KY 40202 USA

**Keywords:** Metabolomics, Missing values, Bayesian, Truncated normal distribution, MAR, MNAR, Markov chain Monte Carlo, Data augmentation

## Abstract

**Background:**

With the rise of metabolomics, the development of methods to address analytical challenges in the analysis of metabolomics data is of great importance. Missing values (MVs) are pervasive, yet the treatment of MVs can have a substantial impact on downstream statistical analyses. The MVs problem in metabolomics is quite challenging and can arise because the metabolite is not biologically present in the sample, or is present in the sample but at a concentration below the lower limit of detection (LOD), or is present in the sample but undetected due to technical issues related to sample pre-processing steps. The former is considered missing not at random (MNAR) while the latter is an example of missing at random (MAR). Typically, such MVs are substituted by a minimum value, which may lead to severely biased results in downstream analyses.

**Results:**

We develop a Bayesian model, called BayesMetab, that systematically accounts for missing values based on a Markov chain Monte Carlo (MCMC) algorithm that incorporates data augmentation by allowing MVs to be due to either truncation below the LOD or other technical reasons unrelated to its abundance. Based on a variety of performance metrics (power for detecting differential abundance, area under the curve, bias and MSE for parameter estimates), our simulation results indicate that BayesMetab outperformed other imputation algorithms when there is a mixture of missingness due to MAR and MNAR. Further, our approach was competitive with other methods tailored specifically to MNAR in situations where missing data were completely MNAR. Applying our approach to an analysis of metabolomics data from a mouse myocardial infarction revealed several statistically significant metabolites not previously identified that were of direct biological relevance to the study.

**Conclusions:**

Our findings demonstrate that BayesMetab has improved performance in imputing the missing values and performing statistical inference compared to other current methods when missing values are due to a mixture of MNAR and MAR. Analysis of real metabolomics data strongly suggests this mixture is likely to occur in practice, and thus, it is important to consider an imputation model that accounts for a mixture of missing data types.

## Background

In many typical high throughput studies, a large number of features (genes/proteins/transcriptomes /metabolites) are measured quantitatively from biological samples, from either humans or animals. Metabolomics is the most downstream field in the omics cascade and provides vital information about metabolic pathways and significant biomarkers related to a certain phenotype. As the downstream products metabolites are very sensitive to various biological states, and they can potentially be used for earlier disease detection compared to other molecular information and further provide contemporaneous information for a variety of other studies [1]. In most mass spectrometry (MS) studies, the number of features is much larger than the number of samples. Because of this large *p* and small *n*, one of the issues is to avoid over-fitting the data. Bayesian methods have become immensely widespread in nearly all scientific fields and this growth is partially attributable to the decrease in the cost of computational costs that are needed to estimate more complex models [2, 3]. There have been several Monte Carlo simulation studies and recent methodologies that have illustrated the benefits of Bayesian methods over frequentist maximum likelihood (ML) methods in situations with small sample sizes [2, 4–6]. Bayesian statistics are used mainly when complex models cannot be estimated using conventional statistics [4], and many complex models use Bayesian methods to avoid likelihood optimization [5]. Bayesian inference typically is not based on large sample asymptotics and can produce more trustworthy results with moderate to small samples, especially when strong prior information is available. By incorporating the prior distributions in model building, one can utilize the initial (un)certainty about a parameter [4].

In addition to small sample sizes, an additional challenge in analyzing metabolomics data is the common occurrence of missing values. Missing values (MVs) in MS can occur from various sources both technical and biological. Taylor, Leiserowitz et al. [7] argue that there are three common sources of missingness in metabolomics studies: i) a metabolite could be truly missing from a sample due to biological reasons, ii) a metabolite can be present in a sample but at a concentration below the detection limit of the MS, and iii) a metabolite can be present in a sample at a level above the detection limit but fail to be detected due to technical issues related to sample processing.

In the statistical literature, missing data can be classified into three categories based on the causality of the missingness [8]: missing completely at random (MCAR) when the missingness is independent of the response, missing at random (MAR) when missingness only depends on the observed responses, and missing not at random (MNAR) when missingness may depend on the unobserved responses. When metabolite abundance is unobserved due to falling below the detection limit, this is MNAR missingness. However, the majority of imputation algorithms for high-throughput data instead exploit the MAR mechanism and use observed values from other genes/proteins/metabolites to impute the MVs. As noted by [9], using any imputation methods for microarray studies in MS omics studies that assume missingness is MCAR or MAR could lead to biased results. However, imputation for MNAR values is fraught with difficulty [7, 10]. While there are a variety MNAR methods in the literature [8, 11], all require assumptions about the relationships between the unobserved values and the probability of observing the value. As the analyst can never see the unobserved values, these assumptions necessarily cannot be confirmed against the observed data. Consequently, it is critical that one use methodology appropriately tailored to account for the sources of MVs in the context of metabolomic analysis.

In this work, we develop a Bayesian model called BayesMetab for the analysis of metabolomics data that systematically accounts for missing values. We allow missingness to be due to either truncation or other technical reasons. Statistical inference is performed by relying on a Markov chain Monte Carlo (MCMC) algorithm that incorporates data augmentation, a common estimation technique in missing value problems [12]. In addition to facilitating parameter estimation, our MCMC algorithm also produces imputed data sets that can used for a variety of purposes (clustering, etc.) beyond the group comparison problem we focus on.

## Methods

### BayesMetab model specification

Here, we describe the full Bayesian specification of our BayesMetab model that including the modeling of the MVs. For sample 1 : *N*, we let *Y*_*i*_ = {*Y*_*i*1_, *Y*_*i*2_, …, *Y*_*iM*_}, be the vector of *M* metabolite intensities. We assume that this vector follows a multivariate normal distribution (possibly after a suitable transformation such as logarithmic): *Y*_*i*_~*N*(*X*_*i*_*β*, Σ), where *X*_*i*_ is the *q*-dimensional design vector for sample *i*, *β* is a *q* × *M* matrix of the regression coefficients. In most cases, the primary goal of inference relates to components of this *β* matrix. For instance, in the common two-group (treatment vs control) problem, we would choose *q* = 2, let the first element of *X*_*i*_ be the intercept and the second element a dummy variable for the treatment group. Differential abundance for metabolite *j* would be captured by the value of *β*_2*j*_. The *M* × *M* covariance matrix Σ captures the dependences between metabolites.

As noted previously, an important component to metabolomics data analysis is handling the MVs. BayesMetab includes a robust approach which both considers MVs due to truncation below the limit of detection (LOD) *ξ* or missing for reasons unrelated to the metabolite abundance (such as other technical failures). Modeling the impact of missing data requires specification of the missing data mechanism (MDM). The MDM is the portion of the model that defines whether or not the value *Y*_*ij*_ is observed and how that depends on the true (sometimes, unobserved) value *Y*_*ij*_. Letting the missingness indicator *R*_*ij*_ be equal to 0 if *Y*_*ij*_ is missing and 1 if *Y*_*ij*_ is observed, our MDM has the form

$$ \Pr \left({R}_{ij}=0\ |{Y}_{ij}\right)=\left\{\begin{array}{c}\alpha, {Y}_{ij}>\xi \\ {}1,{Y}_{ij}\le \xi \end{array}\right.. $$

If the true value of *Y*_*ij*_ is less than the threshold, it will always be missing as the MS platform is unable to detect the magnitude. However, even if *Y*_*ij*_ > *ξ*, there is still a chance that the value *Y*_*ij*_ may be missing, which we assume occurs with common probability *α* across all metabolites j. As this missingness is due to technical reasons unrelated to the abundance of the metabolite, such as poorly plating the sample, assuming a common probability α across all metabolites is reasonable in this case. This missing data mechanism falls into the class of missing not at random (MNAR) since the distribution of *R*_*ij*_ depends on the value of *Y*_*ij*_. With potentially a slight abuse of terminology, we refer to the MDM as consisting of two parts: missingness due to truncation (*Y*_*ij*_ ≤ *ξ*) which we call the MNAR component, and missingness for other technical reasons which we refer to MAR since the probability of observing *Y*_*ij*_ does not depend on the true abundance of the metabolite (except for being above the LOD). As we believe that MVs due to truncation will be based on a LOD shared across all metabolites, we use a common value of ξ for all j. In a context where there is reason to believe that the truncation level should vary by metabolite, it is trivial to extend our approach to allow metabolite-specific LODs.

As a brief detour, we note that a more common selection model [8] allowing MNAR missingness would assume that the logit of *R*_*ij*_ is linearly associated with *Y*_*ij*_: *logit*{Pr(*R*_*ij*_ = 0 | *Y*_*ij*_)} = *α*_0_ + *α*_1_*Y*_*ij*_ . However, this does not represent a reasonable assumption in our context. We know that all observations less than ξ must be missing with probability 1. For values larger than ξ, most believe that the causes of failing to observe this value is unrelated the (unknown) value *Y*_*ij*_, so a linear trend is not appropriate. Due to the lack of biological plausibility of this MDM, we do not consider this choice any further.

A key piece of building the model that can accommodate MV imputation is the choice of the structure of the dependence/covariance. As the dimension of Σ is quite large relative to the sample size *n*, it is important to consider a flexible, lower-dimensional choice for this covariance matrix. To that end, we use the sparse Bayesian infinite factor model due to Bhattacharya and Dunson [13]. This model assume that the covariance matrix can be decomposed using a factor structure Σ = ΛΛ ′  + *D* where *D* is an *M*-dimension diagonal matrix. The Λ matrix of factor loadings has *M* rows and infinitely many columns (in practice, this is truncated to a large value *K*). Model parsimony is achieved by using sparse shrinkage priors for the factor loadings, as well as a constraint guaranteeing that the loadings are stochastically decreasing to zero. There are a few key benefits to using a factor model in our context. First, the factor model represents a reasonable assumption of the dependence between metabolites. As the expression of metabolites are impacted by the joint behavior of various biologic pathways, the latent factors may represent these different pathways and the loading determine which pathways impact which metabolites. Additionally, conditional on the latent factor values, all metabolites are independent, which leads to improved computational performance in the MCMC algorithm in the data imputation step. Finally, the authors [13] provide some theory that guarantees the assumed structure is flexible enough to consistently model any arbitrary covariance structure.

To finalize the model, we use non-informative priors for the remaining parameters. For the MAR missingness probability α, we use a Unif (0, 1) prior. A conjugate normal prior with large variance for the regression coefficients *β* is used,*β*_*kj*_~*N*(0, 100^2^), for all *k* = 1, …, *q*; *j* = 1, …, *M*.

### Model estimation and inference

To fit the BayesMetab model, we develop a computationally efficient Markov chain Monte Carlo algorithm by using a Gibbs sampler that updates each parameter given the current value of the others. This process is repeated for a large number of iterations until convergence to the posterior distribution is achieved.

As mentioned previously, our MCMC algorithm incorporates data augmentation by sampling new values for the missing data within each iteration. By using a latent factor model for Σ, we can equivalently write our model *Y*_*i*_~*N*(*X*_*i*_*β*, Σ), as *Y*_*i*_~*N*(*X*_*i*_*β* + Λ*η*_*i*_, *D*), where *η*_*i*_ is the *K*-vector of latent factor values for sample *i*. Because *D* is diagonal, all *Y*_*ij*_ are independent, conditionally on *η*_*i*_, and we can update each missing *Y*_*ij*_ separately. For each missing *Y*_*ij*_ (those with *R*_*ij*_ = 0), an indicator variable *Z*_*ij*_ is introduced which determines whether *Y*_*ij*_ will be below the LOD threshold *ξ* (*Z*_*ij*_ = 1) or above the threshold (*Z*_*ij*_ = 0). Conditional on the latent factor values, the indicator *Z*_*ij*_ is sampled according to

$$ \Pr \left({Z}_{ij}=1\ |{R}_{ij}=0\ \right)=\frac{\int_{-\infty}^{\xi }{\left(2\pi {\overset{\sim }{\sigma}}_j^2\right)}^{-1/2}\mathit{\exp}\left\{\frac{-1}{2{\overset{\sim }{\sigma}}_j^2}{\left(y-{\overset{\sim }{\mu}}_{ij}\right)}^2\right\}\  dy}{\int_{-\infty}^{\xi }{\left(2\pi {\overset{\sim }{\sigma}}_j^2\right)}^{-1/2}\mathit{\exp}\left\{\frac{-1}{2{\overset{\sim }{\sigma}}_j^2}{\left(y-{\overset{\sim }{\mu}}_{ij}\right)}^2\right\}\  dy+\alpha {\int}_{\xi}^{\infty }{\left(2\pi {\overset{\sim }{\sigma}}_j^2\right)}^{-1/2}\mathit{\exp}\left\{\frac{-1}{2{\overset{\sim }{\sigma}}_j^2}{\left(y-{\overset{\sim }{\mu}}_{ij}\right)}^2\right\}\  dy}, $$

where ${\overset{\sim }{\mu}}_{ij}$ is the *j*^*th*^ element of *X*_*i*_*β* + Λ*η*_*i*_ and ${\overset{\sim }{\sigma}}_j^2$ is the (*j*, *j*) element of *D*. This probability represents how likely the metabolite’s MV is to be due to truncation (MNAR component) versus other sources (MAR component) based on the mean value of the metabolites (from the regression structure) and the information contained in the latent factor from the related and observed metabolites. As the integrals required are all probabilities under the normal distribution, these can be efficiently evaluated. Given *Z*_*ij*_, the value of the missing *Y*_*ij*_ is sampled by

$$ Z=1:{Y}_{ij}\sim \mathrm{Truncated}\ \mathrm{Normal}\ \left({\overset{\sim }{\mu}}_{ij},{\overset{\sim }{\sigma}}_j^2\right)I\left(-\infty, \xi \right) $$

$$ Z=0:{Y}_{ij}\sim \mathrm{Truncated}\ \mathrm{Normal}\ \left({\overset{\sim }{\mu}}_{ij},{\overset{\sim }{\sigma}}_j^2\right)I\left(\xi, \infty \right) $$

After running this data augmentation step for each missing value, we have a complete dataset which may be used to update the other parameters.

Conditionally on *η*_*i*_ factor values, we sample the regression coefficients independently for each metabolite. We let *β*_*m*_ be the *p* vector of coefficients for metabolite *m*, H be the *K × N* matrix of *η*_*i*_, ${\sigma}_m^2$ be the (*m, m*) element of the *D* covariance matrix, and Λ_*m*_ be the *K* vector of factor loadings for metabolite *m*. It follows that ${Y}_m^{\ast }={Y}_m-{\Lambda}_m\mathrm{H}\sim N\left(X{\beta}_m,{\sigma}_m^2{I}_N\right)$. We update *β*_*m*_ by sampling from ${N}_p\left({\left({\Omega}^{-1}+{\sigma}_m^{-2}{X}^{\prime }X\right)}^{-1}{X}^{\prime }{Y}_m^{\ast },{\left({\Omega}^{-1}+{\sigma}_m^{-2}{X}^{\prime }X\right)}^{-1}\right)$ where Ω is the *p × p* prior covariance matrix.

To sample the parameters of the covariance matrix, we follow the required steps from Bhattacharya and Dunson [13] as described in the Additional file 1: Supplemental Material. Conditional on the full data set, the MAR parameter α can be conjugately sampled from

$$ \alpha \sim \mathrm{Beta}\left({\sum}_{ij}\left[\left(1-{R}_{ij}\right)I\left({Y}_{ij}>\xi \right)\right]+1,{\sum}_{ij}\left[{R}_{ij}I\left({Y}_{ij}>\xi \right)\right]+1\right). $$

After a sufficient number of iterations, the MCMC chain provides a useful summary of the posterior distribution of the parameters. Convergence and mixing of the MCMC sample is typically assessed by evaluating the trace plots, autocorrelation, and/or Geweke statistics for the regression coefficients (and other parameters). In the examples run in the following sections, we have found 2500 iterations to be sufficient to provide adequate mixing and convergence to the posterior distribution. In addition to using the posterior samples of *β* for inference, we may also extract one or more of the imputed datasets to analyze using standard methods for complete data, such as a two-sample t-test.

### Simulation study

The simulations were conducted with 100 replications and are similar in spirit to those used in Tutz and Ramzan [14]. For each replication we generated data with different combinations of sample sizes *n* and number of metabolites *M*. Each set of metabolites for a given sample were drawn from a *M* dimensional multivariate normal distribution with a mean vector *μ* and a blockwise correlation matrix Σ. The means of the metabolites are assumed to be different and are generated from a Uniform(−5, 5) distribution. The differentially abundant metabolites (the first 100 out of the total) have mean abundance one unit larger in one group. For the smaller sample size (10 samples) we also incorporated an effect size of 1.6 for the first 100 metabolites. In the correlation matrix, a block-tridiagonal structure was used with blocks consisting of correlation *ρ* = 0.8 and 0.4; the variance was 1 for all metabolites. For the proportion of MVs, three levels were studied: 9% missing, 15% missing and 30% missing. Missing data were created based on our two kinds of missingness, MNAR and MAR. Within each level of missing, a one-third and two-third combination was used to create both MNAR and MAR. We looked at the scenario where MNAR is greater than MAR, MNAR is less than MAR, only MAR and only MNAR. For example in 9% missing, we considered 6% of values to be missing due to MNAR and 3% due to MAR. The LOD *ξ* was chosen to be the sample percentile needed to produce the specified MNAR percent, and the remaining MVs are chosen at random from the response greater than *ξ*. After inducing missingness, each dataset was passed through a cleaning process where metabolites with more than 50% missing observations within either group were eliminated.

We carried out a simulation study to compare the performance of the methods by first evaluating the estimation error. We compared the bias and the mean squared error (MSE) for the *β* regression coefficients based on the Bayesian method and other approaches such as zero, mean and minimum imputation, KNN truncation and GSimp. Zero, mean and minimum are standard replacement approaches where the MV is replaced with zero, the sample mean and the sample minimum for the respective metabolite in each group. GSimp was developed by Wei et al. [15] and is a MV imputation method based on left-censored (MNAR) using an iterative Gibbs sampler approach which allows flexible choice of the threshold/truncation value. KNN truncation was developed by Shah et al. [16] which was a modified algorithm of the KNN method that uses the estimates of a truncated normal distribution to impute MVs.

After single dataset imputation under these approaches, a general linear regression was fit to the complete imputed dataset to estimate the *β*, and hypothesis testing was performed through the usual two-sample t-test on the imputed data. We consider two implementations of BayesMetab method. BayesInf considers inference using the posterior sample of the parameters from the MCMC chain. Estimates of *β* are taken as the mean of the posterior sample, and hypothesis testing is carried out by comparing the ratio of the posterior mean and the posterior standard deviation (a Z-score like quantity) to the standard normal distribution. Additionally, we perform the standard two-group t-test using the final imputed data set, labeled BayesImp, to match with the framework employed for the other imputation methods. We then evaluate the power, type I error and the area under the ROC curve (AUC) using the *p* values from the tests to compare the approaches.

### Real data study

To further compare methodology, we applied our approach to in vivo metabolomics data on myocardial infarction (MI) [17]. The data consisted of two groups, MI vs control, 5 samples in each group and 288 metabolites. Adult mice were subjected to permanent coronary occlusion (myocardial infarction; MI) or Sham surgery. The study was aimed to examine the metabolic changes that occur in the heart in vivo during heart failure using mouse models of permanent coronary ligation. The MI group had 220 metabolites with complete values, 6 metabolites with complete missing and 62 metabolites had 4.8% missing values whereas the controls had 241 metabolites with complete values, 7 metabolites with complete missing and 40 metabolites had 7.8% missing values. The data was screened so that we only considered metabolites with at least three out of five observed in each group, leaving 263 metabolites under consideration. For the Bayesian approach, the LOD for this dataset is considered as the minimum value of the dataset as commonly used in untargeted metabolomics, and GSimp uses the default of per-metabolite minimum. Details of the experiments are described in Sansbury et al. [17]. For easiest comparison, we use our BayesImp approach, GSimp and KNN-Truncation to create a single imputed dataset and compare the groups using the two-sample t-test.

## Results

### Simulation study results

**Fig. 1:**
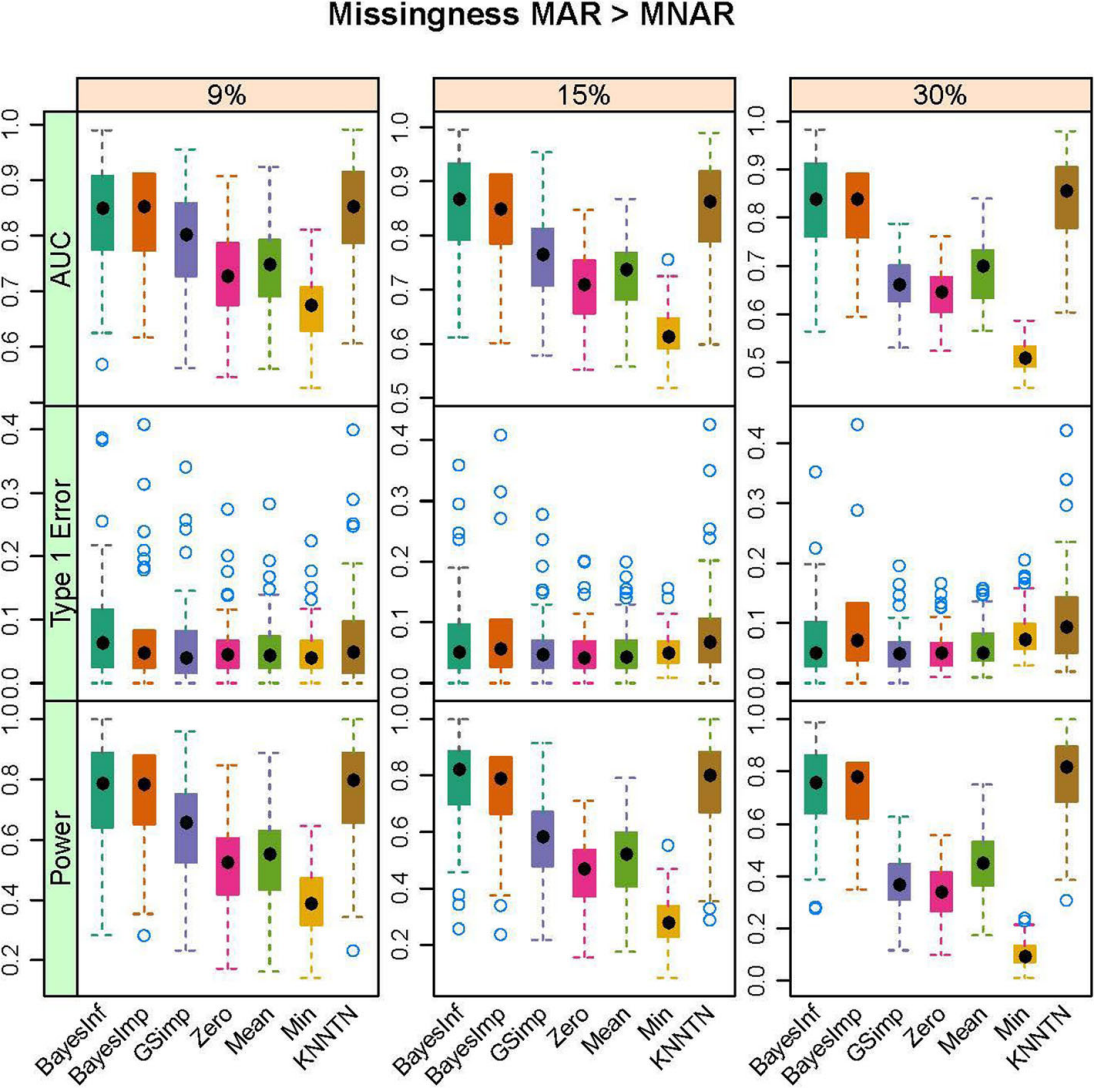
Box plots for Power, Type 1 Error and AUC for Bayesian, GSimp, Zero, Min, Mean and KNNTN methods for 100 datasets, 30 samples by 225 metabolites. Total missing was considered at 9, 15, and 30% and within each missing MNAR is less than MAR.

**Fig. 2:**
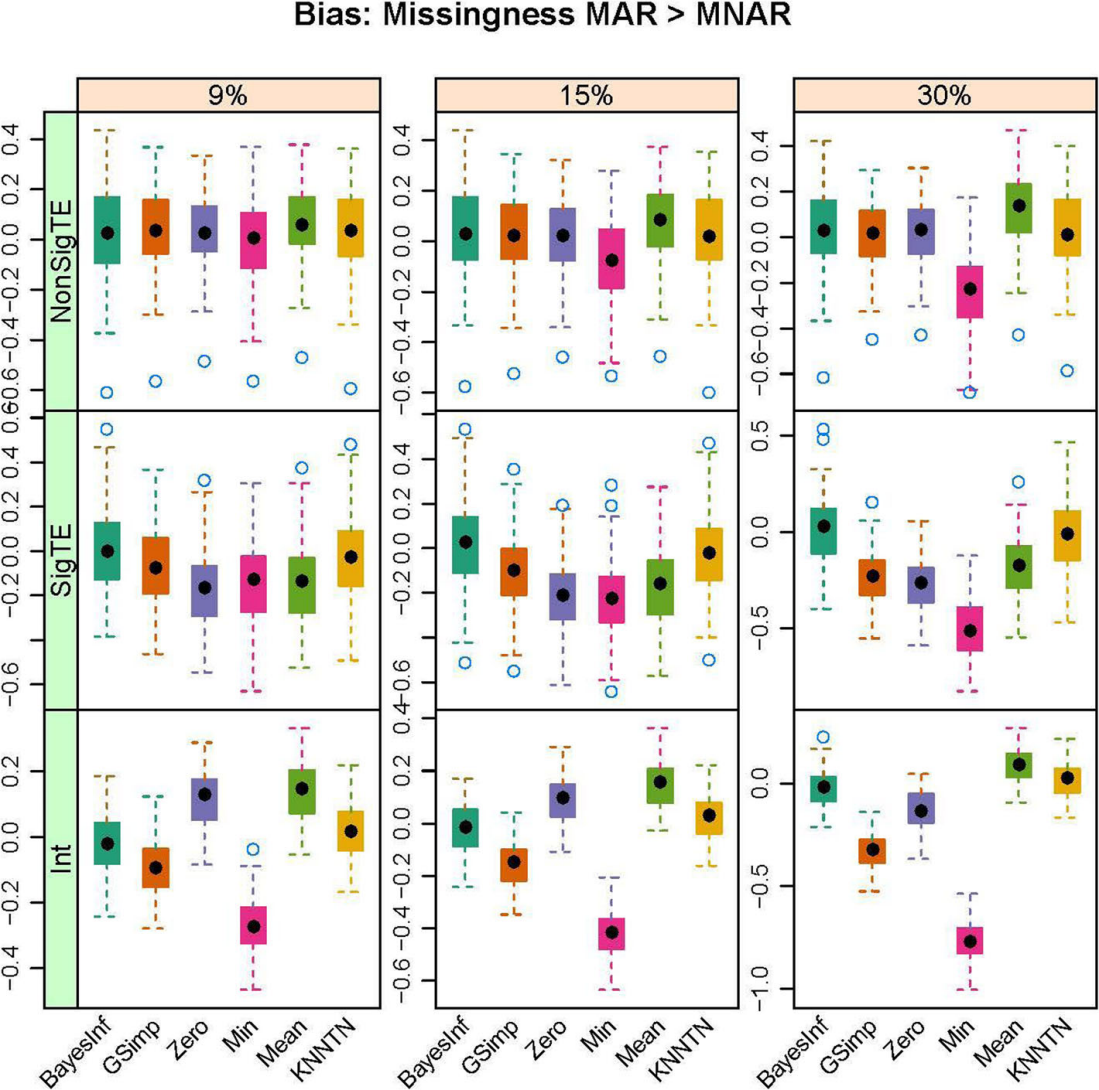
Box plots for Bias for Bayesian, GSimp, Zero, Min, Mean and KNNTN methods for 100 datasets, 30 samples by 225 metabolites. Total missing was considered at 9, 15, and 30% and within each missing MNAR is less than MAR.

**Fig. 3:**
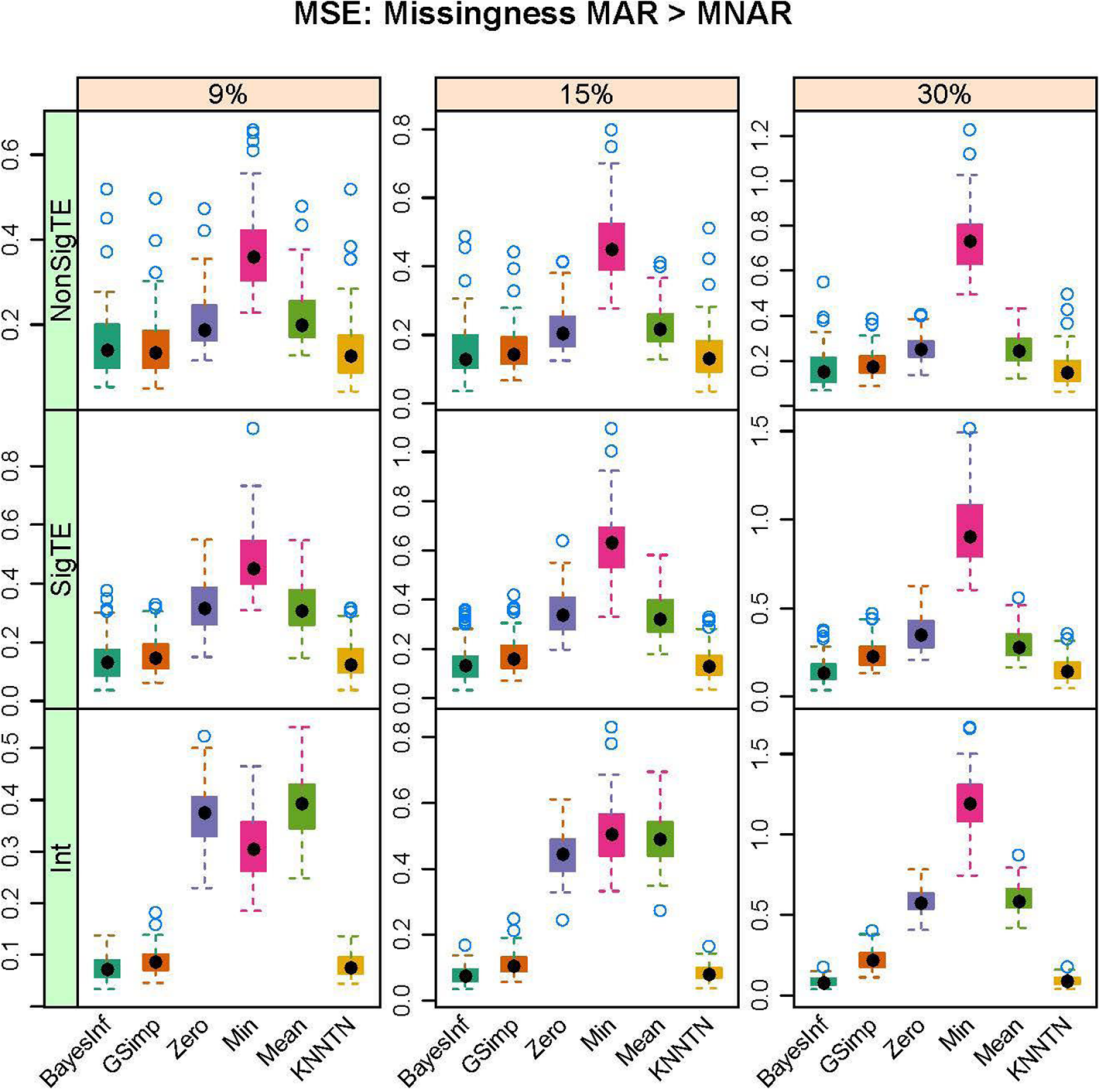
Box plots for MSE for Bayesian, GSimp, Zero, Min, Mean and KNNTN methods for 100 datasets, 30 samples by 225 metabolites. Total missing was considered at 9, 15, and 30% and within each missing MNAR is less than MAR.

**Fig. 4:**
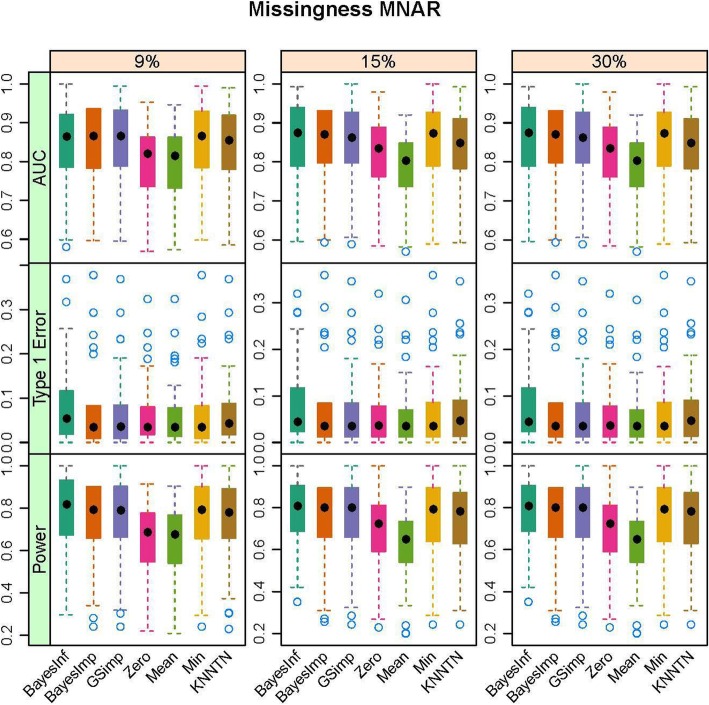
Box plots for Power, Type 1 Error and AUC for Bayesian, GSimp, Zero, Min, Mean and KNNTN methods for 100 datasets, 30 samples by 225 metabolites. Total missing was considered at 9, 15, and 30% and completely MNAR

**Fig. 5:**
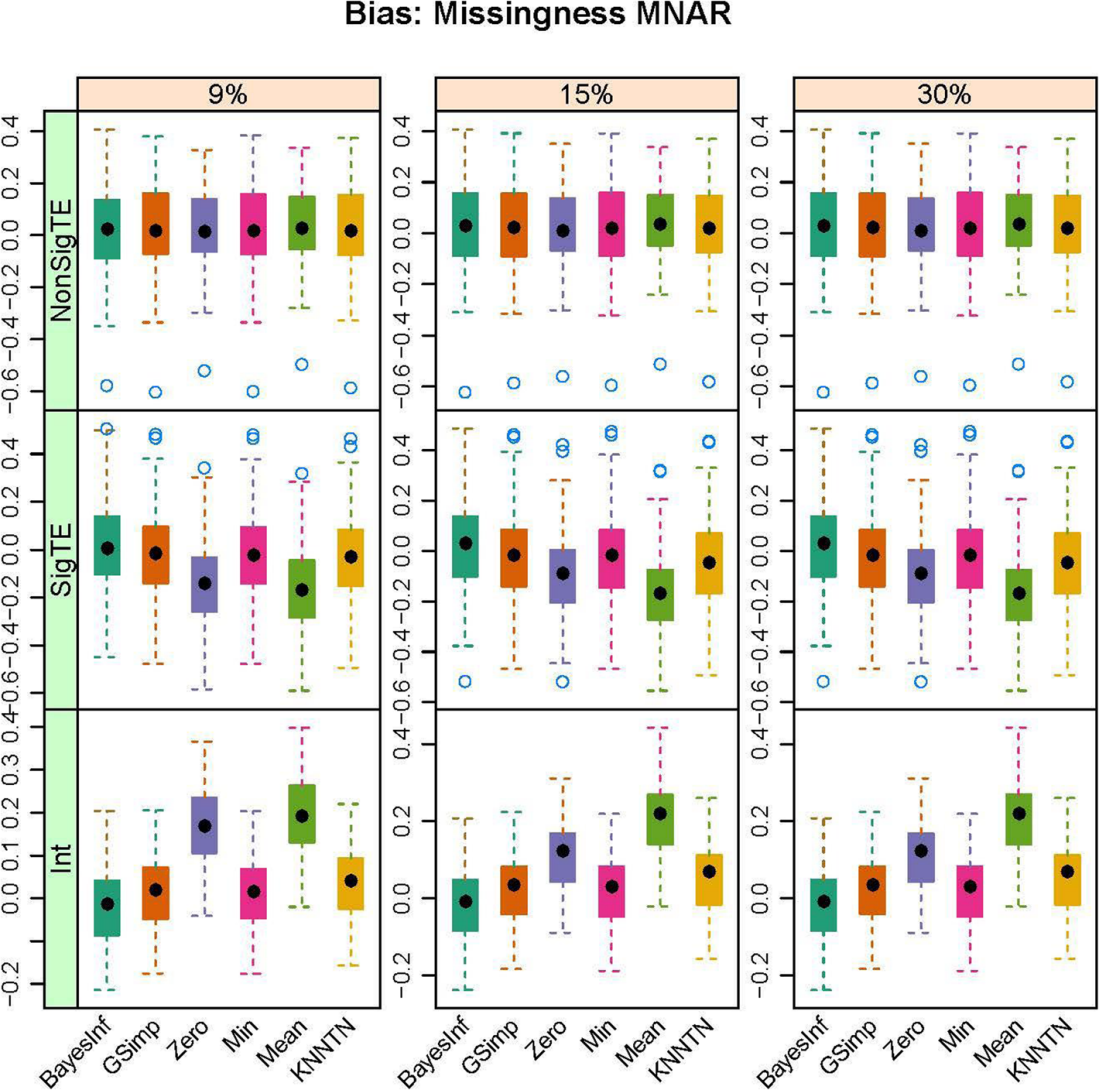
Box plots for Bias for Bayesian, GSimp, Zero, Min, Mean and KNNTN methods for 100 datasets, 30 samples by 225 metabolites. Total missing was considered at 9, 15, and 30% and completely MNAR

**Fig. 6:**
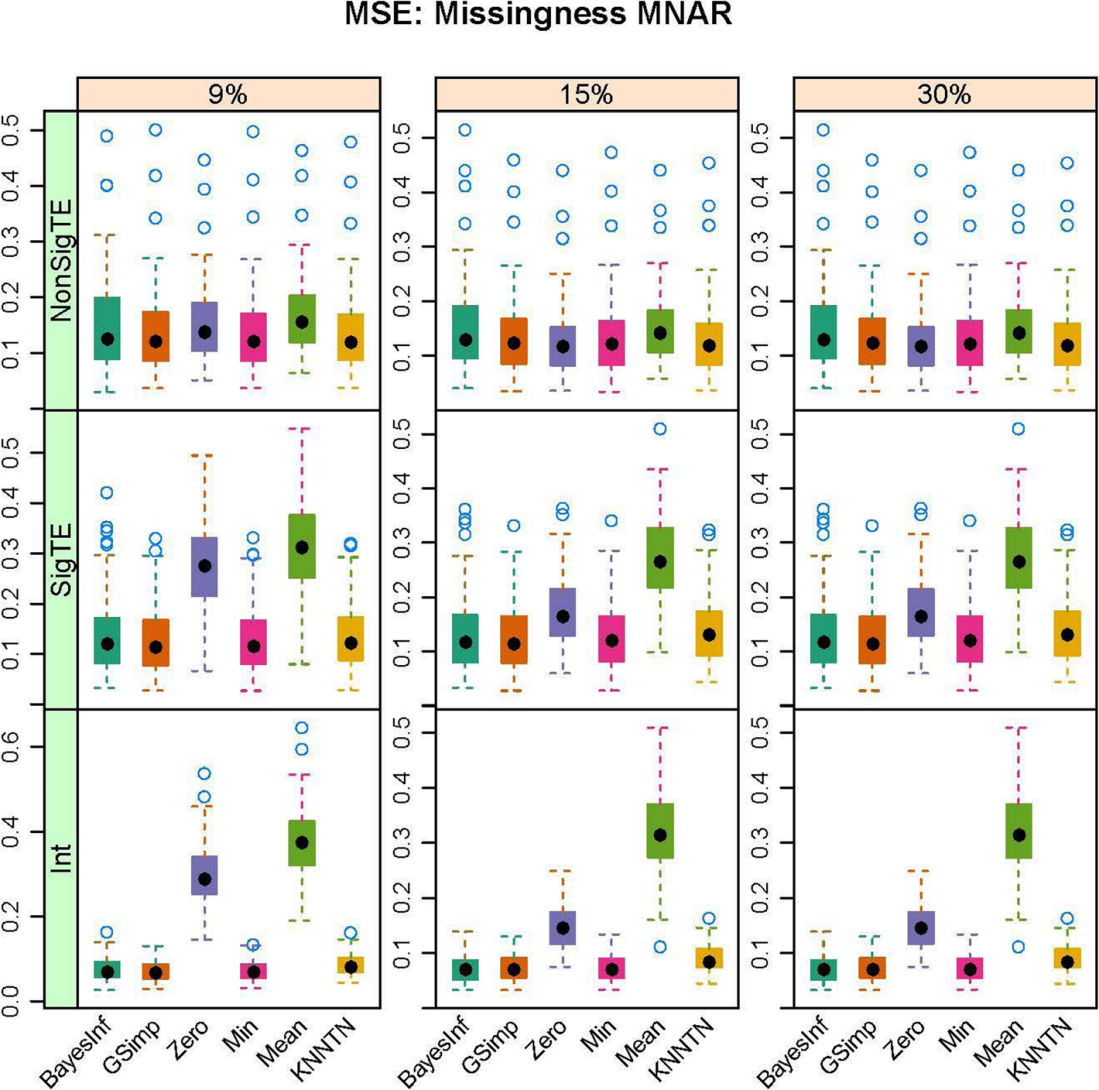
Box plots for MSE for Bayesian, GSimp, Zero, Min, Mean and KNNTN methods for 100 datasets, 30 samples by 225 metabolites. Total missing was considered at 9, 15, and 30% and completely MNAR

In this section, we present the results of the simulation studies comparing the performance of BayesMetab with the other approaches. The simulations were based on three sample sizes: 10 samples by 200 metabolites, 30 samples by 225 metabolites and 50 samples by 400 metabolites. Within each sample size, 4 different types of missingness was considered: mixture where MAR < MNAR, mixture where MAR > MNAR, MAR only, and MNAR only. Figure 1 shows the power, type1 error and the AUC for the competing methods in the 30 samples by 225 metabolites data when 1/3 of the MVs are below the LOD (MNAR) and 2/3 above (MAR). *P*-values were computed based on the standard t-test and the power, type 1 error and area under the curve were computed. The AUC and power are both higher for BayesMetab method (BayesInf and BayesImp) relative to GSimp. This is expected in this scenario, as GSimp is restricted to imputing values to the left tail of the distributions. The separation between the methods increases as the missing rate increases, with BayesImp only having a slightly elevated type-I error rate at 30% missing. We further considered the estimation accuracy for the intercept and the treatment effect across both differentially abundant and non-differentially abundant metabolites. While the bias for estimating the beta coefficients is relatively similar between the two approaches (Fig. 2), our BayesImp method has a smaller MSE than GSimp particularly as the percent missing increases (Fig. 3). Our previously developed KNN-TN method has comparable power to the BayesImp and BayesInf methods, though the error rate seems to exceed the nominal 5% threshold as the percent missing hits 30%. As expected, the naïve simple imputation methods (zero, mean, and min) perform poorly in all these scenarios. In the scenario where all missingness is due to intensities below the LOD (MNAR only), all the methods (except the naïve mean and zero approaches) demonstrate comparable power, type I error, and AUC (Fig. 4), as well as similar bias (Fig. 5) and MSE (Fig. 6) for estimating the beta coefficients. The separation between our method and GSimp becomes greatest with 100% MAR (Additional file 2: Figures S1-S3) and is slightly lower when MNAR > MAR (Additional file 2: Figures S4-S6), generally following a decreasing trend with increasing percent MNAR. Similar results hold for the 50 samples by 400 metabolites simulations (Additional file 2: Figures S7-S18). Due to the low power with 10 samples, we increase the effect size to 1.6. In this scenario, the AUC and power are both higher for BayesMetab method (BayesInf and BayesImp) relative to GSimp and comparable to KNN-TN method. When MAR > MNAR, BayesMetab outperforms KNN-TN with 9 and 15% missingness on power and AUC. The type 1 error for BayesMetab is lower than the KNN-TN and with 30% missingness the type 1 error for KNN-TN is also higher. Similar results hold when missingness is completely MNAR and when MAR < MNAR when comparing BayesMetab with KNN-TN (Additional file 2: Figures S19-S30).

### Real data study results

**Fig. 7:**
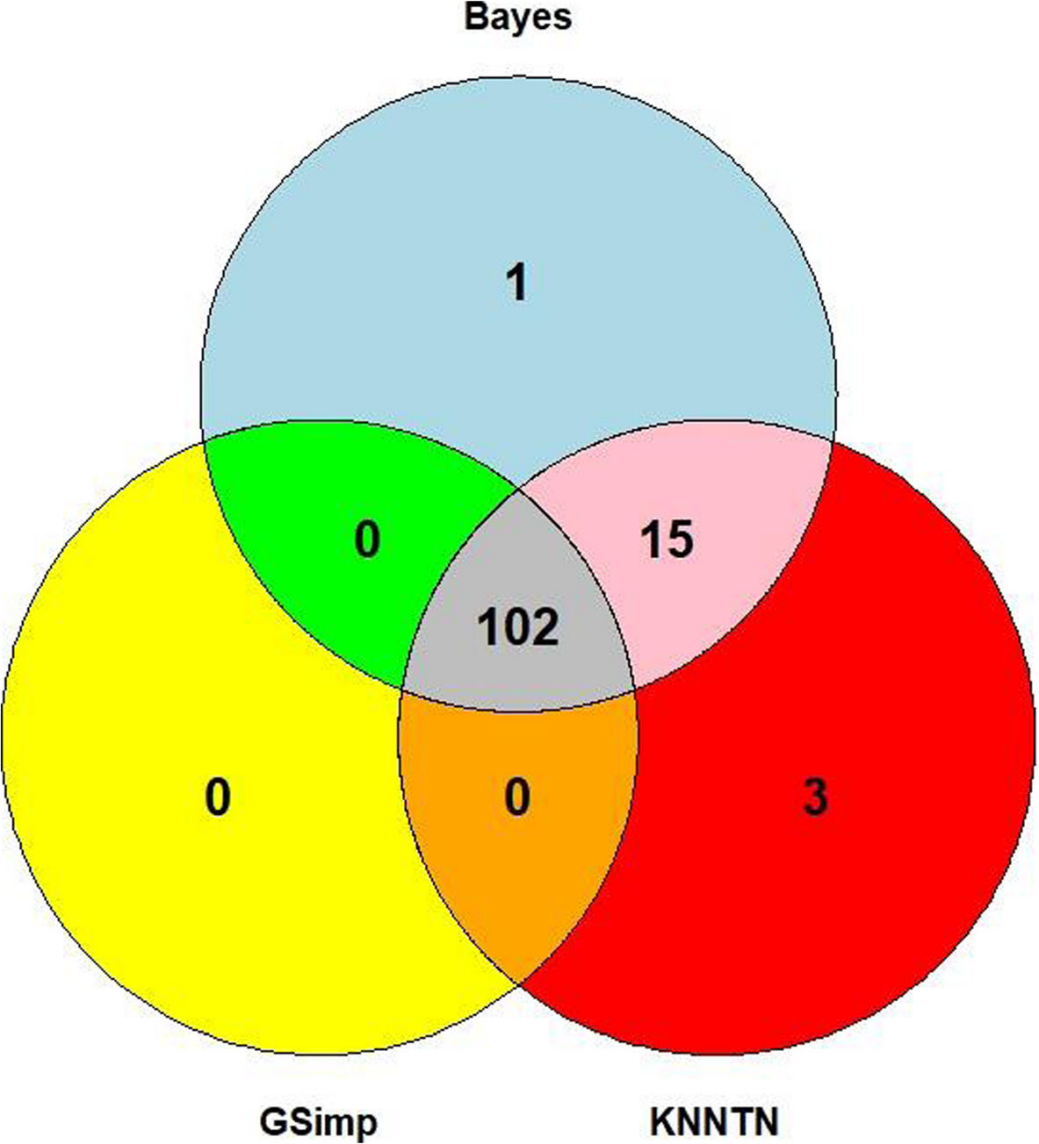
Venn diagram to show the differences between the significant metabolites detected by BayesMetab, GSimp and KNN-Truncation. Note that 91 of 102 shared discoveries are from metabolites with complete data

Using the imputed dataset from the various methods, we conducted an unpaired t-test to identify the number of significant metabolites based on a significance level of 0.05. Figure 7 gives a comparison of the number of significant metabolites found by BayesMetab, GSimp and KNN Truncation, and the number of commonalities. Note that 91 of the discoveries come from metabolites with fully observed data in both groups, so these do not represent differences in the methodologies. Further, all of the metabolites flagged as differentially abundant by GSimp are also detected by our BayesImp, but the Bayesian approach finds an addition 16 metabolites of interest when compared with GSimp. In this data, we find the BayesMetab and KNN-TN tend to perform similarly.

**Fig. 8:**
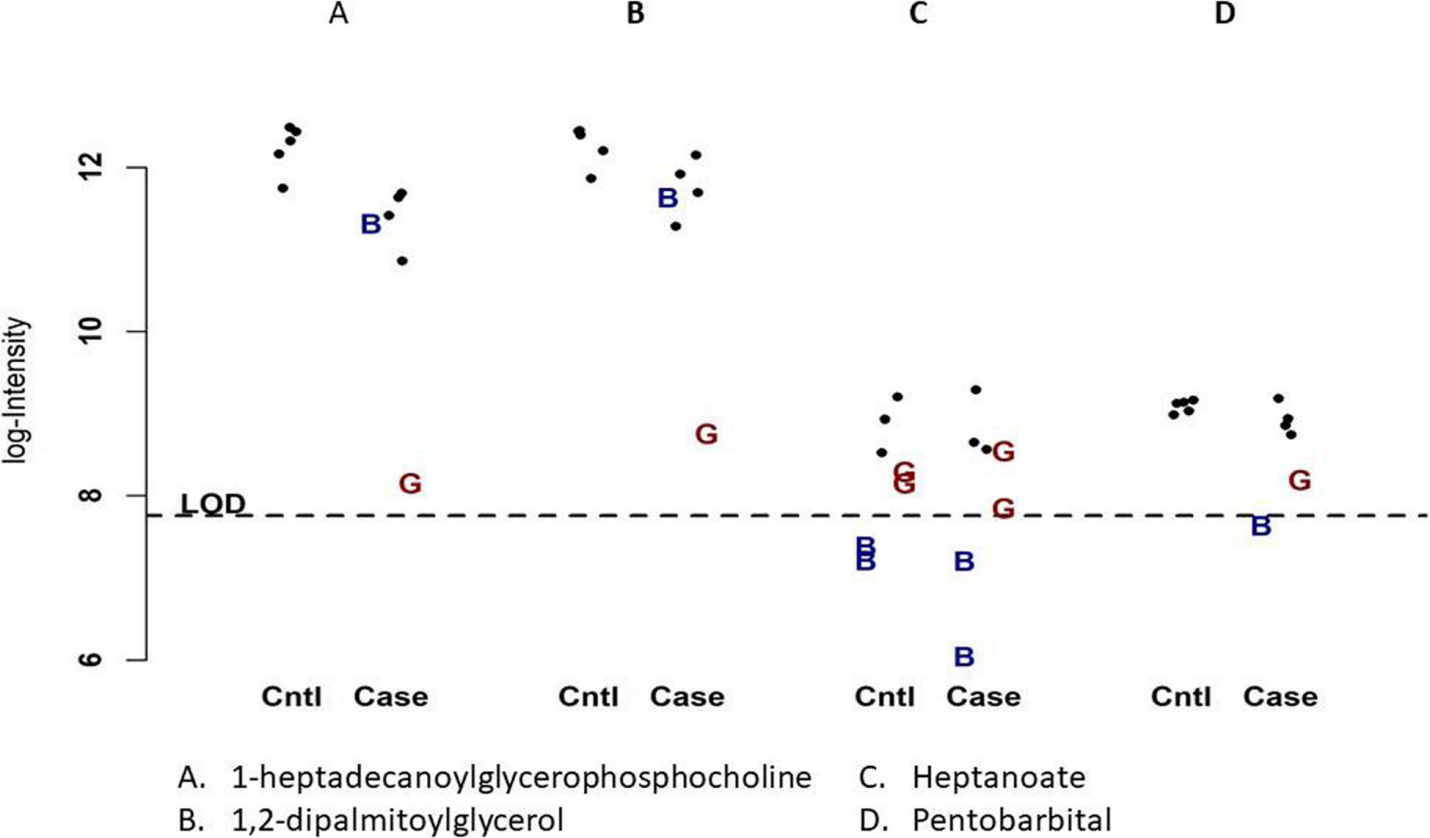
Comparison of imputed missing values imputed from the Bayesian and GSimp method for 4 metabolites. The x-axis represents the cases and controls of the each of the metabolites, and the y-axis represents the log intensity values. The dark black circle represent the observed values and the “B” and “G” represent missing values imputed by the Bayesian and GSimp method respectively. The horizontal line represents the LOD of the data

We further looked at the distributions of those 16 metabolites that were significant with BayesMetab method and not GSimp to see how the imputed values are imputed based on the BayesMetab and GSimp method. Figure 8 represents the distribution of, 1,2-dipalmitoylglycerol 1-heptadecanoylglycerophosphocholine, heptanoate and pentobarbital from the MI study data respectively. The horizontal line represents the LOD, the “G” represent the values imputed by the GSimp method and the “B” represent the values imputed by the Bayesian method. In metabolites A and B, GSimp tends to impute MVs to be substantially lower and outside of the range of the observed values, leading to inflated standard deviations and lost power. Most of the metabolites from the 16 unique significant metabolites from the Bayesian method followed the similar distribution as shown in the two examples above.

To further understand the role of imputation near the LOD, we evaluated the distribution of two metabolites, heptanoate (**C**) and pentobarbital (**D)**. These metabolites were not significant based on both methods and have values close to the LOD. Here, BayesMetab suspects these MVs are more likely to be due to truncation than in panels (A) and (B) since the observed values are near *ξ*, and the imputed values are below the LOD. For the most part, BayesMetab approach does a similar job to GSimp of imputing these MVs.

While comparing the results with Sansbury et al. [16], Sansbury et al. performed a metabolomic analysis using the half minimum imputation method and found 87 of the 288 metabolites analyzed to be significantly different based on an unpaired t-test. Of the 288 metabolites measured, 41 and 24% of the metabolites were lipids and amino acids. Table 1 shows the significant metabolites uniquely identified by the BayesMetab and KNN-TN as compared to the GSimp method. The majority of the significant metabolites in Table 1 represent the lipid super pathway and lysolipid sub pathway. As seen in the Supplemental Table II by Sansbury et al. 34 out of the 87 significant metabolites were within the lipid pathway. Since the additional significant metabolites identified by the Bayesian method also represent the lipid and lysolipid pathways, this indicates that BayesMetab method may be correctly imputing these values as the metabolites within the lipid pathway are closely affiliated with myocardial infarction.

**Table 1:** Significant metabolites uniquely identified by the BayesMetab and KNN-TN and its super pathway and sub pathway metabolism. Note ** were metabolites identified by the KNN-TN method

Metabolite	SUPER_PATHWAY	SUB_PATHWAY
1,2-dipalmitoylglycerol	Lipid	Diacylglycerol
1-heptadecanoylglycerophosphocholine	Lipid	Lysolipid
1-linoleoylglycerophosphocholine	Lipid	Lysolipid
1-palmitoleoylglycerophosphocholine*	Lipid	Lysolipid
1-palmitoylglycerophosphoethanolamine	Lipid	Lysolipid
1-palmitoylglycerophosphoinositol*	Lipid	Lysolipid
1-pentadecanoylglycerophosphocholine*	Lipid	Lysolipid
2-arachidonoylglycerophosphocholine*	Lipid	Lysolipid
2-linoleoylglycerophosphocholine*	Lipid	Lysolipid
2-linoleoylglycerophosphoethanolamine*	Lipid	Lysolipid
2-oleoylglycerophosphocholine*	Lipid	Lysolipid
4-hydroxybutyrate (GHB)	Lipid	Fatty acid, monohydroxy
7-alpha-hydroxycholesterol	Lipid	Sterol/Steroid
phosphopantetheine	Cofactors /vitamins	Pantothenate and CoA metabolism
prostaglandin I2	Lipid	Eicosanoid
sarcosine (N-Methylglycine)	Amino acid	Glycine, serine and threonine metabolism
Squalene**	Lipid	Sterol / Steroid
2-palmitoylglycerol**	Lipid	Monoacylglycerol
2-palmitoleoylglycerophosphocholine**	Lipid	Lysolipid

## Discussion

The purpose of this study was to develop a Bayesian approach for imputing missing values in metabolomics. When metabolites are below the detection limit of the instrumentation, it is considered to missing not at random. In contrast, missing values resulting from technical errors unrelated to the metabolite abundance are considered missing at random. To this end, we introduce our BayesMetab model that incorporates data augmentation and includes a parameter which allows MVs to occur via either the MNAR (below the limit of detection) or MAR mechanisms. Since MNAR is due to the detection limit, we consider the detection limit as a truncation point and assume that the observed metabolites follow a truncated normal distribution. We evaluated BayesMetab method with other recently developed imputation methods for truncated metabolomics data (GSimp, KNN-TN) [15, 16] as well as traditionally used naïve approaches which take a simplistic approach to imputation (zero, mean, and minimum imputation). Our simulation results revealed superior performance of our methodology compared to the other imputation approaches when missingness was due to a mixture of MAR and MNAR data or MAR alone, and competitive results when missingness was completely MNAR. Our analysis of metabolomics data from a mouse myocardial infarction study revealed that our approach identified several additional metabolites relative to GSimp with differential abundance between the control (sham surgery) and experimental (permanent coronary occlusion) groups that were categorized as lipids and lysolipids and were of direct biological relevance.

In our simulation study, we evaluated our algorithm in the scenario of untargeted metabolomics datasets where we assumed missingness could arise based on either MAR and MNAR situations. We used the minimum value as the lower truncation point and compared our results with those from GSimp, KNN-TN and other simple imputation approaches. GSimp was originally developed in the context of targeted metabolomics where each metabolite has its own truncation level, but their code allows the user to select a left-censoring value (such as overall minimum for LOD trunctation or -∞ for MAR). Throughout, we have use the default of selecting the minimum observed value for each metabolite. However, this may not be a reasonable choice for untargeted metabolomics, as the design of the mass spectrometers is such that the LOD is common across all metabolites. However, if we select the left-censoring value to be the LOD, then all MVs will be imputed below this LOD. This will perform very poorly in situations like (A) and (B) from Fig. 8. In practice, we strongly believe that most untargeted metabolomics datasets consist a mixture of missingness, and thus it is important to consider an imputation model that can capture both. When comparing BayesMetab with KNN-TN, the KNN-TN method has higher type 1 error rates and BayerMetab outperforms. One of the key differences is that KNN-TN uses a non-parametric method.

In our simulation study we investigated data from a normal distribution, whereas in many cases metabolite data may be non-normally distributed. In these cases we suggest to first transform the data to normality (e.g., using a log or Box-Cox transform) and then apply BayesMetab to impute the values. However, our current study lacks a comprehensive evaluation of these imputation algorithms using a diverse set of real experimental data to determine the true impact on downstream statistical analyses [18]. To that end, our future work will focus on simulating data based on real studies (e.g., from the Metabolomics Workbench [19]) using a simulation approach which mimics the underlying multivariate distribution of the data [20]). Importantly, this approach permits us to simulate missing values which accounts for the LOD in real data sets and thus can easily incorporate both MNAR and MAR values. In a recently completed study evaluating a number of MV imputation algorithms designed for either MAR or MNAR data on a variety of data sets, results revealed that random forest imputation performed best for MAR data while GSimp was optimal for MNAR data [21]. However, the real issue is that data from metabolomics (and other) studies are likely to be a mixture of both MAR and MNAR data, and practitioners will have a difficult time deciding a priori which imputation algorithm to use when faced with this choice. Our methodology naturally adapts to estimate the percentage of MAR vs. MNAR data and incorporates this information into the imputation estimates. While having a clear advantage over other imputation algorithms for imputing simulated data with a mixture of missing due to MAR and MNAR, we have also shown evidence in real metabolomics data that this mixture exists and is an important consideration for finding relevant results from a metabolomics study.

## Conclusions

In conclusion, BayesMetab is a comprehensive approach for imputing high dimensional data where there is missingness partially due to a truncation (detection) threshold. Results based on simulated data show that BayesMetab method generally has higher power for detecting differentially abundant metabolites compared to the other imputation algorithms when there is both missing at random and missing due to a threshold value. This is accompanied by a concomitant reduction in MSE for the parameter estimates from the linear model. Due to our model’s adaptive nature, when data are missing completely due to MNAR our results remain competitive with specialized MNAR-only algorithms. Inspection of missing and imputed values in metabolomics data from a mouse myocardial infarction study indicate that a mixture of MAR and MNAR values is highly plausible, and reanalysis of this data using our approach revealed several statistical significant metabolites not previously identified that were of direct biological relevance to the study. Our approach can further be applied to other high-dimensional data sets that contain a mixture of missing values due to MNAR (below a threshold value) and MAR, for instance delta-CT values from qRT-PCR array cards [22] and proteomics data.

## Supplementary information

**Additional file 1.** Supplemental material to briefly discuss the sparse Bayesian infinite factor model by Bhattacharya and Dunson [13] that we use to model the dependence structure of the metabolite data.

**Additional file 2.** Supplementary Figures. S1 – S30

## Data Availability

The datasets analysed during the current study are available from supplementary materials of the study by Sansbury et al. (2014): https://www.ahajournals.org/doi/suppl/10.1161/CIRCHEARTFAILURE.114.001151. R code for simulations and real data analysis are available from the corresponding author on reasonable request. The authors are developing an R package to implement the methods presented in the paper.

## Abbreviations

AUC: Area under the curve
KNN: k nearest neighbor
KNN-TN: k nearest neighbor truncation
LOD: Limit of detection
MAR: Missing at random
MCAR: Missing completely at random
MCMC: Markov Chain Monte Carlo
MDM: Missing data mechanism
MI: Myocardial Infarction
MNAR: Missing not at random
MS: Mass Spectrometry
MSE: Mean squared error
MVs: Missing values
