# Supplementary material for: BayesMetab: treatment of missing values in metabolomic studies using a Bayesian modeling approach
Source: BMC Bioinformatics. 2019 Dec 20;20(Suppl 24):673. doi: 10.1186/s12859-019-3250-2 (PMC6923847; doi:10.1186/s12859-019-3250-2)
Supplement: Supplementary file 1 — Additional file 1. Supplemental material to briefly discuss the sparse Bayesian infinite factor model by Bhattacharya and Dunson [13] that we use to model the dependence structure of the metabolite data. [file 12859_2019_3250_MOESM1_ESM.docx]

**Supplemental Material**

In this supplemental material document, we briefly discuss the sparse Bayesian infinite factor model by Bhattacharya and Dunson [13] that we use to model the dependence structure of the metabolite data. As noted in the manuscript, we can equivalently write $Y_{i} \sim N\left( X_{i}\beta, \Sigma\right)$ as $Y_{i} \sim N\left( X_{i}\beta+ \Lambda\eta_{i}, D \right)$ where $\eta_{i}$ is the $K$-vector of latent factor values for sample$i$, Λ is a $pxK$ matrix of factor loadings/coefficients, and D is a diagonal matrix with variance beyond that explained by the regression and factor structures.

Briefly, the covariance model proposed in [13] involves hierarchy

$$\lambda_{mh}| \phi_{mh}, \tau_{h} \sim N\left( 0, \phi_{mh}^{-1}\tau_{h}^{-1} \right), \phi_{mh} \sim Ga \left( \nu/2,\nu/2 \right), \tau_{h}=\prod_{l=1}^{h} \delta_{1},$$

$$\delta_{1} \sim Ga \left( a_{1},1 \right), \delta_{l} \sim Ga \left( a_{2},1 \right), l\geq2, \sigma_{m}^{-2}\sim Ga\left( a_{\sigma}, b_{\sigma} \right) \left( m=1,\ldots, M \right),$$

where $\delta_{l}=\left( l=1,\ldots, \infty\right),$are independent, $\tau_{h}$ is a global shrinkage parameter for the *h* th column and the $\phi_{mh}$s are the local shrinkage parameters for the elements in the *h* th column. The hyperparameters $a_{1}$ and $a_{2}$ are also given Gamma (2, 1) priors. In our analyses, we let$\nu=3$,$a_{\sigma}=1$, and$b_{\sigma}=0.3$.

As noted in [13], this model can be sampled by iterating through the following steps. We adjust notation from [13] to match our use.

**Step 1:** If we denote the $m^{th}$ row of $\Lambda_{K}$ by$\lambda_{m}^{T}$, then $\lambda_{m}$s have independent conditionally conjugate posteriors,

$$\pi\left( \lambda_{m} \right|- ) \sim N_{K}\left\{ \left( D_{m}^{-1}+\sigma_{m}^{-2}\eta^{T}\eta\right)^{-1}\eta^{T}\sigma_{m}^{-2}y^{\left( m \right)},\left( D_{m}^{-1}+\sigma_{m}^{-2}\eta^{T}\eta\right)^{-1} \right\},$$

where$\eta=\left( \eta_{1},\ldots,\eta_{n} \right)^{T}$, $D_{m}^{-1}=\mathrm{diag}\left( \phi_{m1}\tau_{1},\ldots,\phi_{mk^{*}}\tau_{k^{*}} \right)$ and$y^{*(m)}=Y_{m}-X\beta_{m}$. Given the other parameters, $\pi\left( \lambda_{m} \right|- )$ denotes the conditional posterior of$\lambda_{m}$.

**Step 2:** Sample $\sigma_{m}^{-2}, m=1,\ldots, M$ from conditionally independent posteriors

$$\pi\left( \sigma_{m}^{-2} \right|- ) \sim Ga \left\{ a_{\sigma}+\frac{n}{2}, b_{\sigma}+\frac{1}{2}\sum_{i=1}^{n} \left( y_{ij}-X_{i}\beta_{m}-\lambda_{m}^{T}\eta_{i} \right)^{2} \right\}$$

**Step 3:** Sample$\eta_{i}, i=1,\ldots,n$, from conditionally independent posteriors

$$\pi\left( \eta_{i} |- \right) \sim N_{K}\left\{ \left( I_{K}+{\Lambda^{T}\Sigma}^{-1}\Lambda\right)^{-1}\Lambda_{k^{*}}^{T}\Sigma^{-1}{y_{i}}^{*},\left( I_{K}+{\Lambda^{T}\Sigma}^{-1}\Lambda\right)^{-1} \right\}$$

**Step 4:** Sample$\phi_{mh}$ from

$$\pi\left( \phi_{mh} |- \right) \sim Ga \left( \frac{\nu+1}{2},\frac{\nu+\tau_{h}\lambda_{mh}^{2}}{2} \right)$$

**Step 5:** Sample $\delta_{1}$ from

$$\pi\left( \delta_{1}|- \right) \sim Ga \left( a_{1}+\frac{MK}{2}, 1+\frac{1}{2}\sum_{l=1}^{K} \tau_{l}^{(1)}\sum_{m=1}^{M} \phi_{ml}\lambda_{ml}^{2} \right)$$

and for $h \geq2,$ sample $\delta_{h}$ from

$$\pi\left( \delta_{h}|- \right) \sim Ga \left( a_{2}+\frac{M}{2}\left( K-h+1 \right), 1+\frac{1}{2}\sum_{l=h}^{K} \tau_{l}^{(h)}\sum_{m=1}^{M} \phi_{ml}\lambda_{ml}^{2} \right)$$

where $\tau_{l}^{\left( h \right)}= \prod_{t=1, t\neq h}^{l} \delta_{t} \mathrm{for}h=1,\ldots,K.$

**Step 6:** Update $a_{1}$ and $a_{2}$ using a Metropolis-Hastings step within the Gibbs sampler.
