# Supplementary material for: BayesMetab: treatment of missing values in metabolomic studies using a Bayesian modeling approach
Source: BMC Bioinformatics. 2019 Dec 20;20(Suppl 24):673. doi: 10.1186/s12859-019-3250-2 (PMC6923847; doi:10.1186/s12859-019-3250-2)
Supplement: Supplementary file 2 — Additional file 2. Supplementary Figures. S1 – S30 [file 12859_2019_3250_MOESM2_ESM.docx]

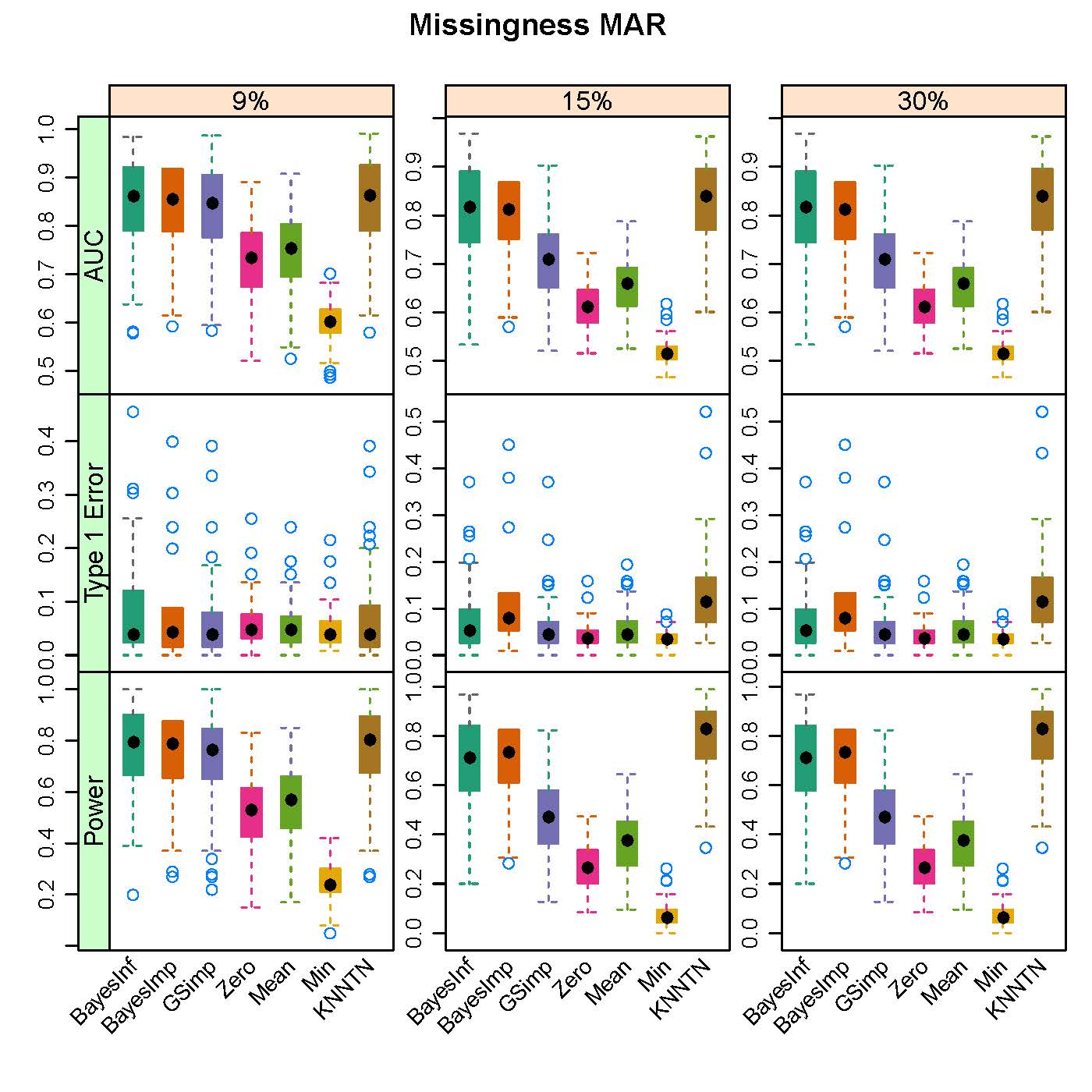


**Figure S1**: Box plots for Power, Type 1 Error and AUC for Bayesian, GSimp, Zero, Min, Mean and KNNTN methods for 100 datasets, 30 samples by 225 metabolites. Total missing was considered at 9%, 15%, and 30% and completely MAR.


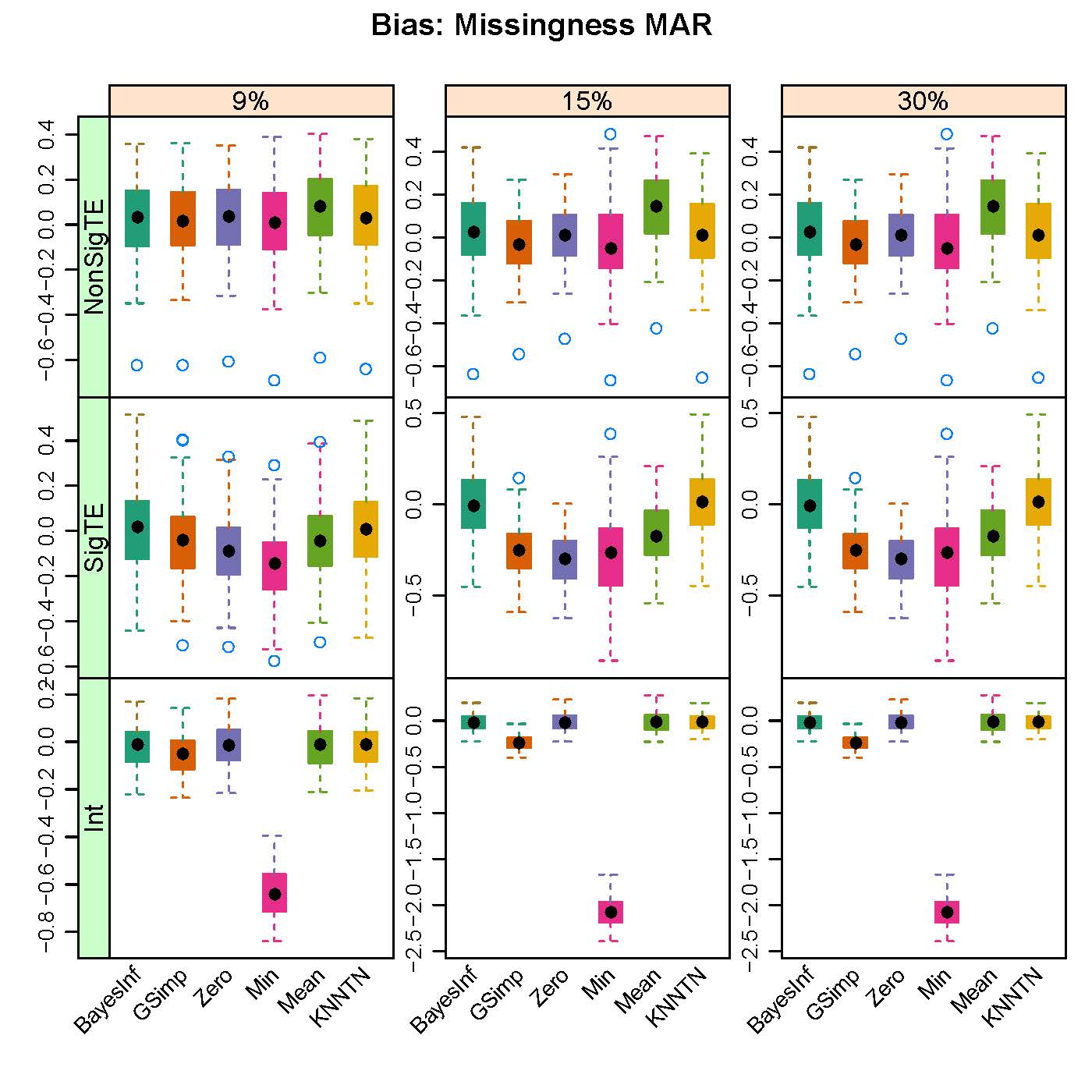


**Figure S2**: Box plots for Bias for Bayesian, GSimp, Zero, Min, Mean and KNNTN methods for 100 datasets, 30 samples by 225 metabolites. Total missing was considered at 9%, 15%, and 30% and completely MAR.


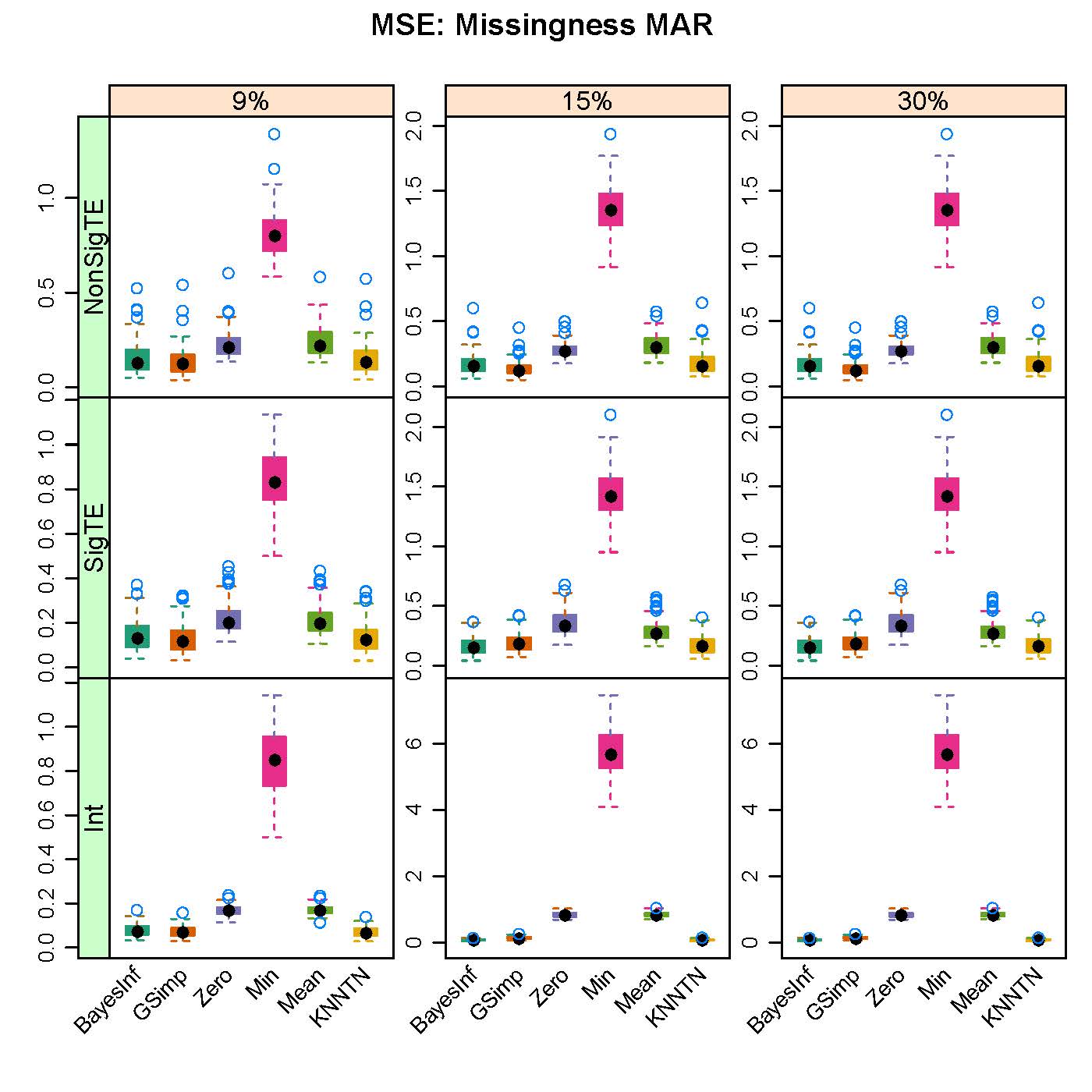


**Figure S3**: Box plots for MSE for Bayesian, GSimp, Zero, Min, Mean and KNNTN methods for 100 datasets, 30 samples by 225 metabolites. Total missing was considered at 9%, 15%, and 30% and completely MAR.

**
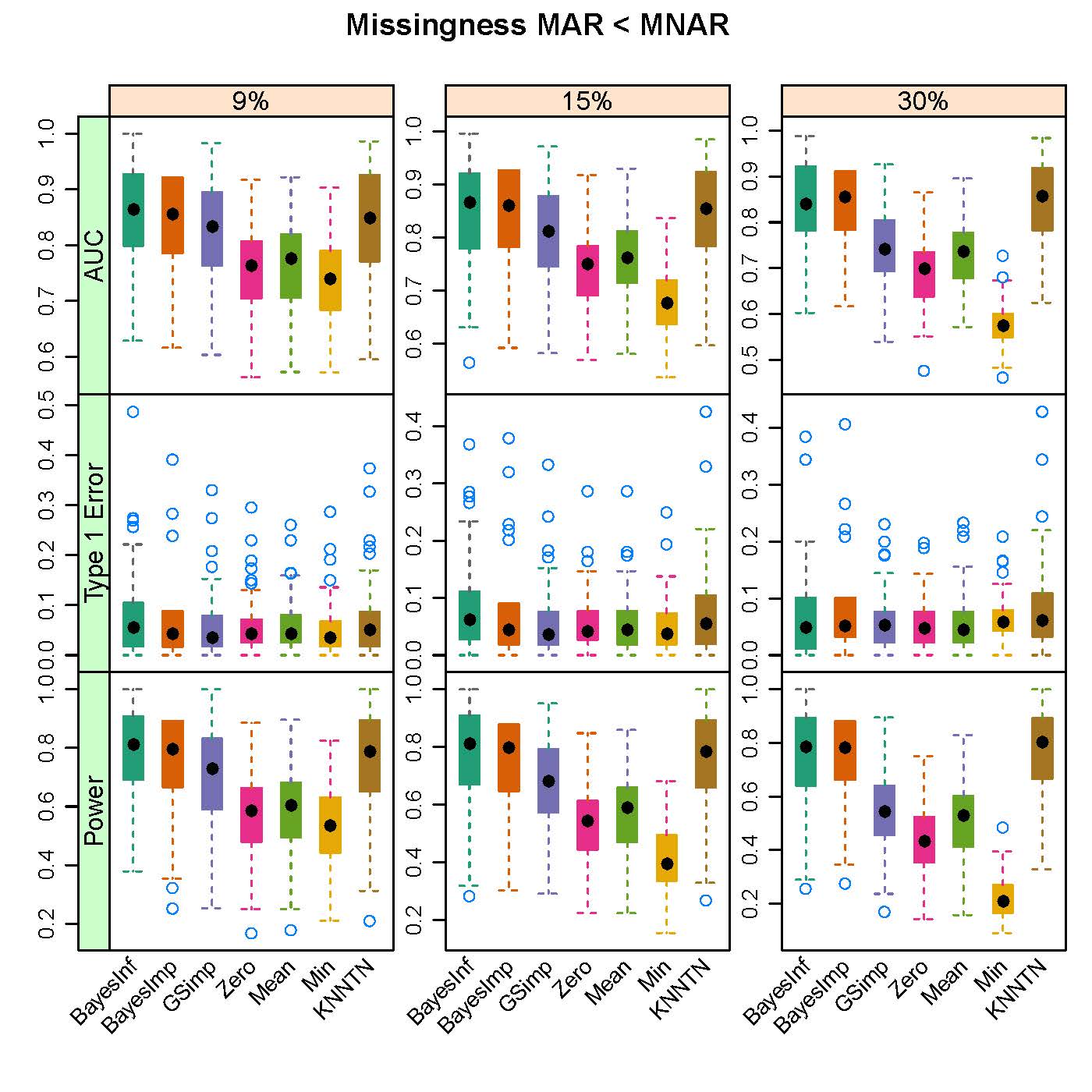
**

**Figure S4**: Box plots for Power, Type 1 Error and AUC for Bayesian, GSimp, Zero, Min, Mean and KNNTN methods for 100 datasets, 30 samples by 225 metabolites. Total missing was considered at 9%, 15%, and 30% and within each missing MNAR is greater than MAR.


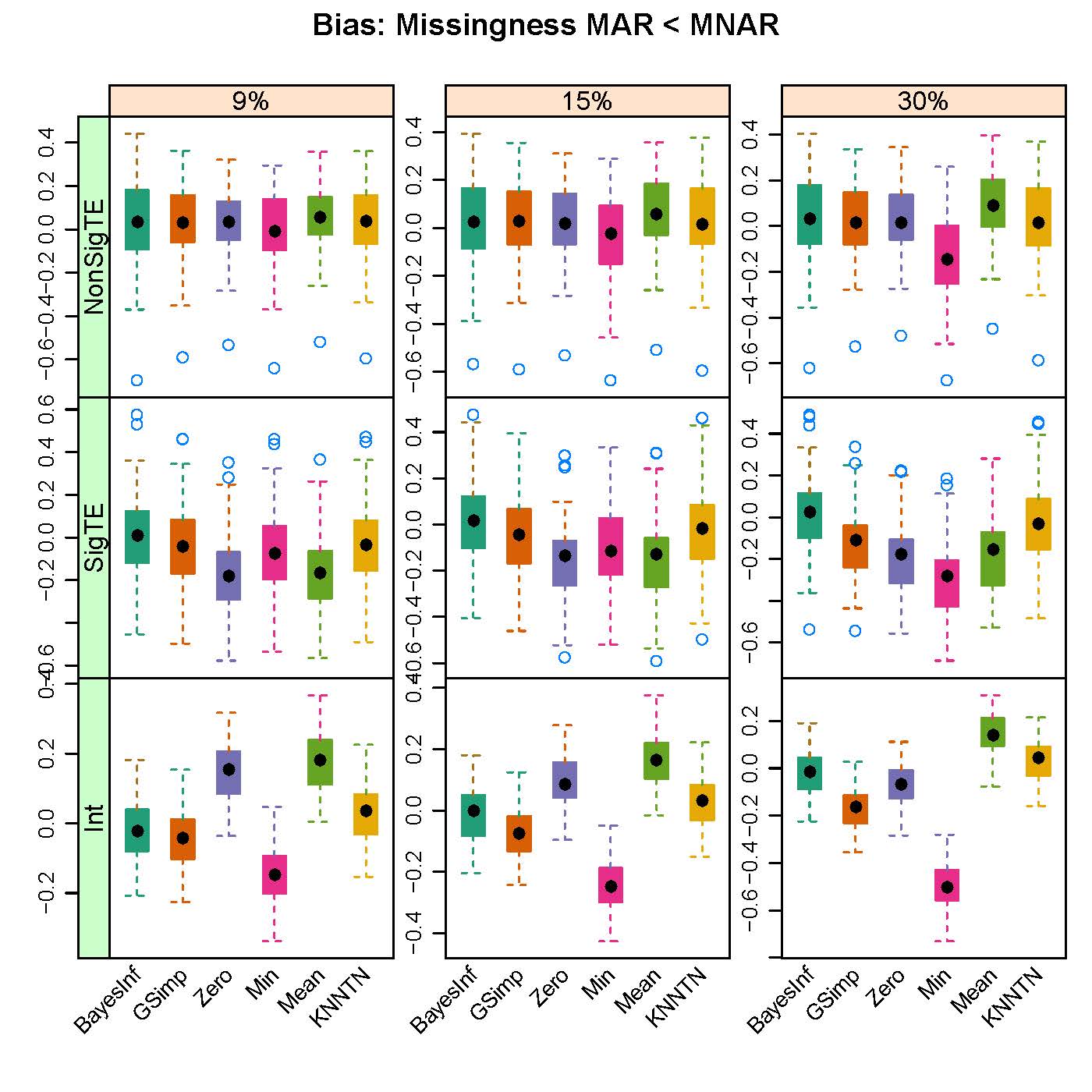


**Figure S5**: Box plots for Bias for Bayesian, GSimp, Zero, Min, Mean and KNNTN methods for 100 datasets, 30 samples by 225 metabolites. Total missing was considered at 9%, 15%, and 30% and within each missing MNAR is greater than MAR.


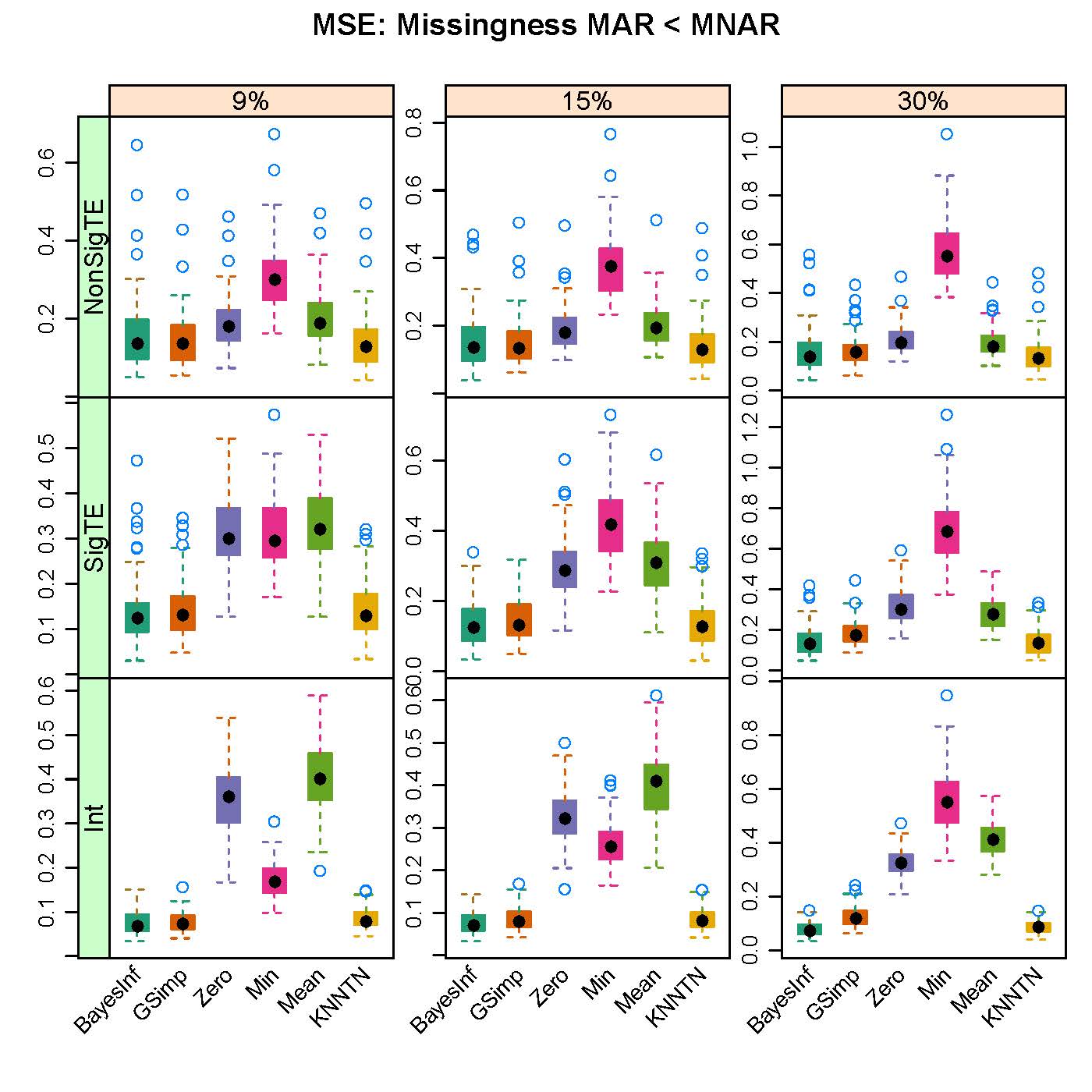


**Figure S6**: Box plots for MSE for Bayesian, GSimp, Zero, Min, Mean and KNNTN methods for 100 datasets, 30 samples by 225 metabolites. Total missing was considered at 9%, 15%, and 30% and within each missing MNAR is greater than MAR.

**
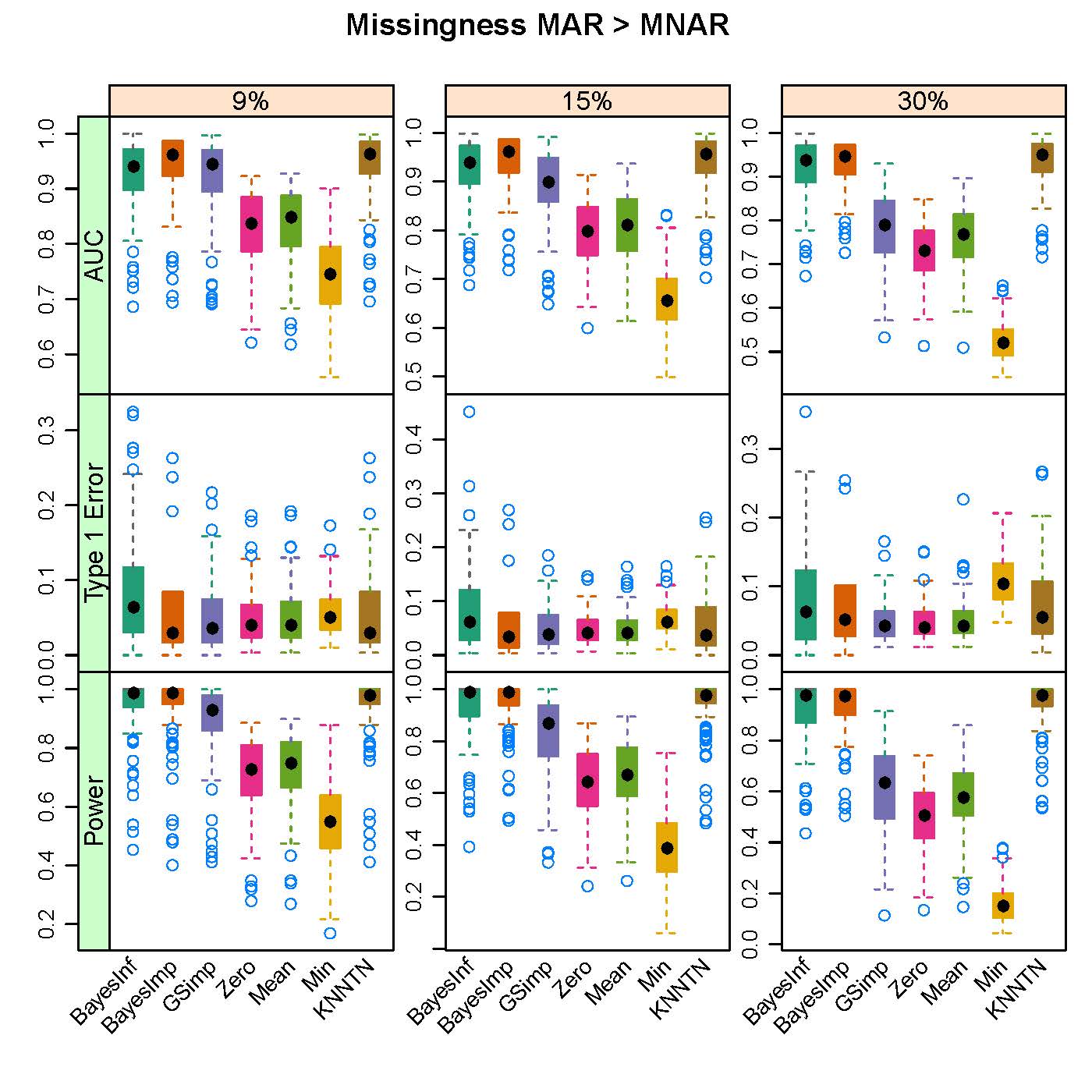
**

**Figure S7**: Box plots for Power, Type 1 Error and AUC for Bayesian, GSimp, Zero, Min, Mean and KNNTN methods for 100 datasets, 50 samples by 400 metabolites. Total missing was considered at 9%, 15%, and 30% and within each missing MNAR is less than MAR.


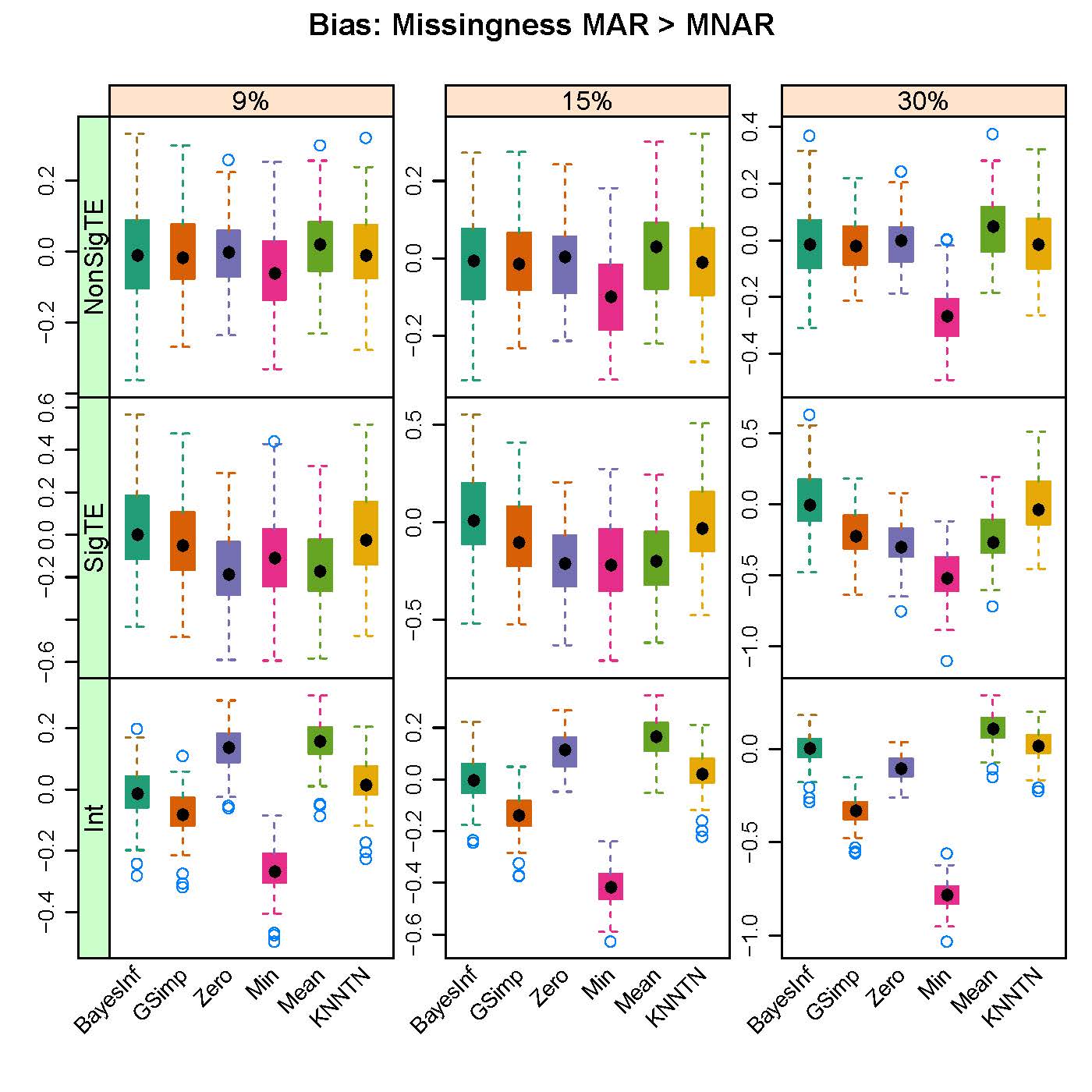


**Figure S8**: Box plots for Bias for Bayesian, GSimp, Zero, Min, Mean and KNNTN methods for 100 datasets, 50 samples by 400 metabolites. Total missing was considered at 9%, 15%, and 30% and within each missing MNAR is less than MAR.


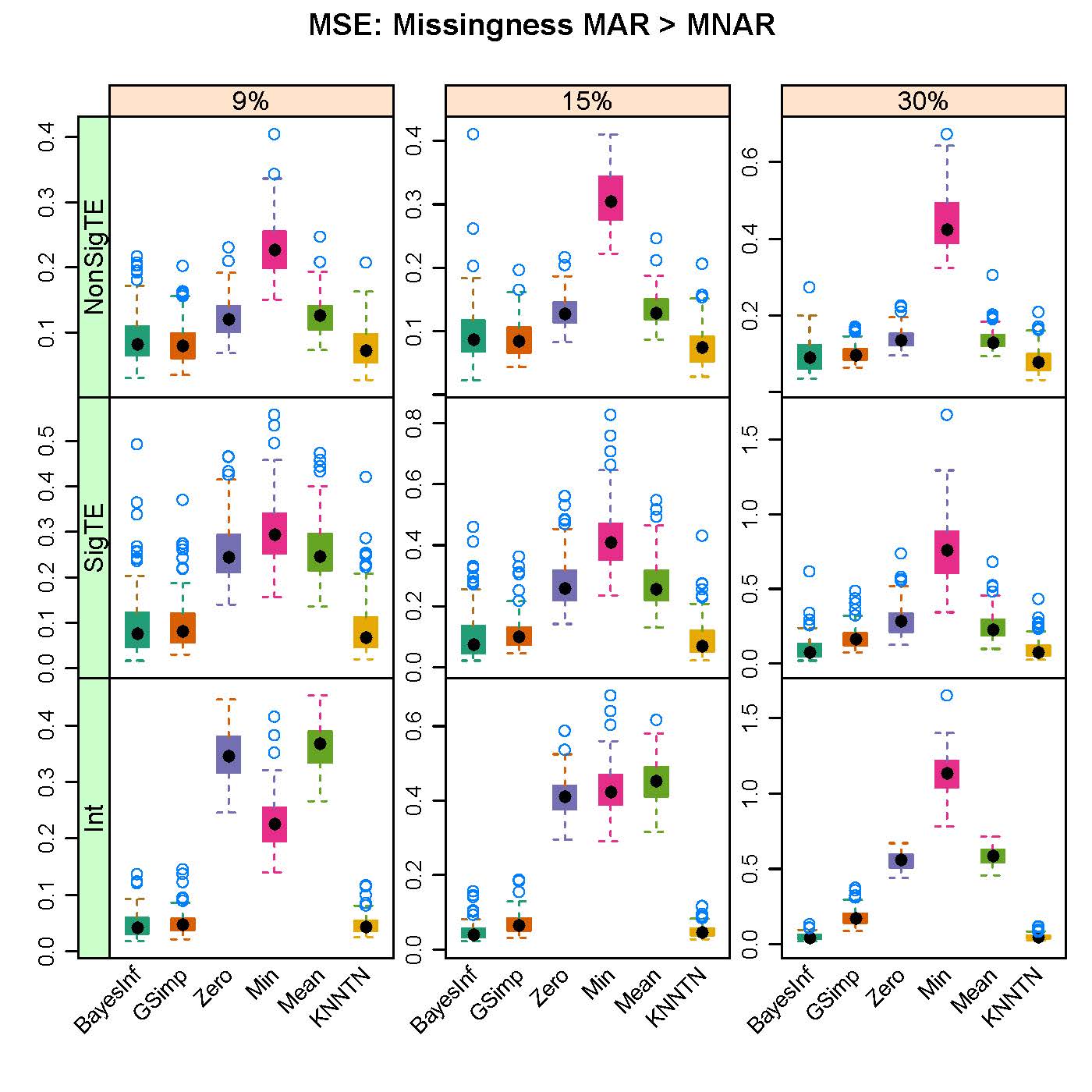


**Figure S9**: Box plots for MSE for Bayesian, GSimp, Zero, Min, Mean and KNNTN methods for 100 datasets, 50 samples by 400 metabolites. Total missing was considered at 9%, 15%, and 30% and within each missing MNAR is less than MAR.

**
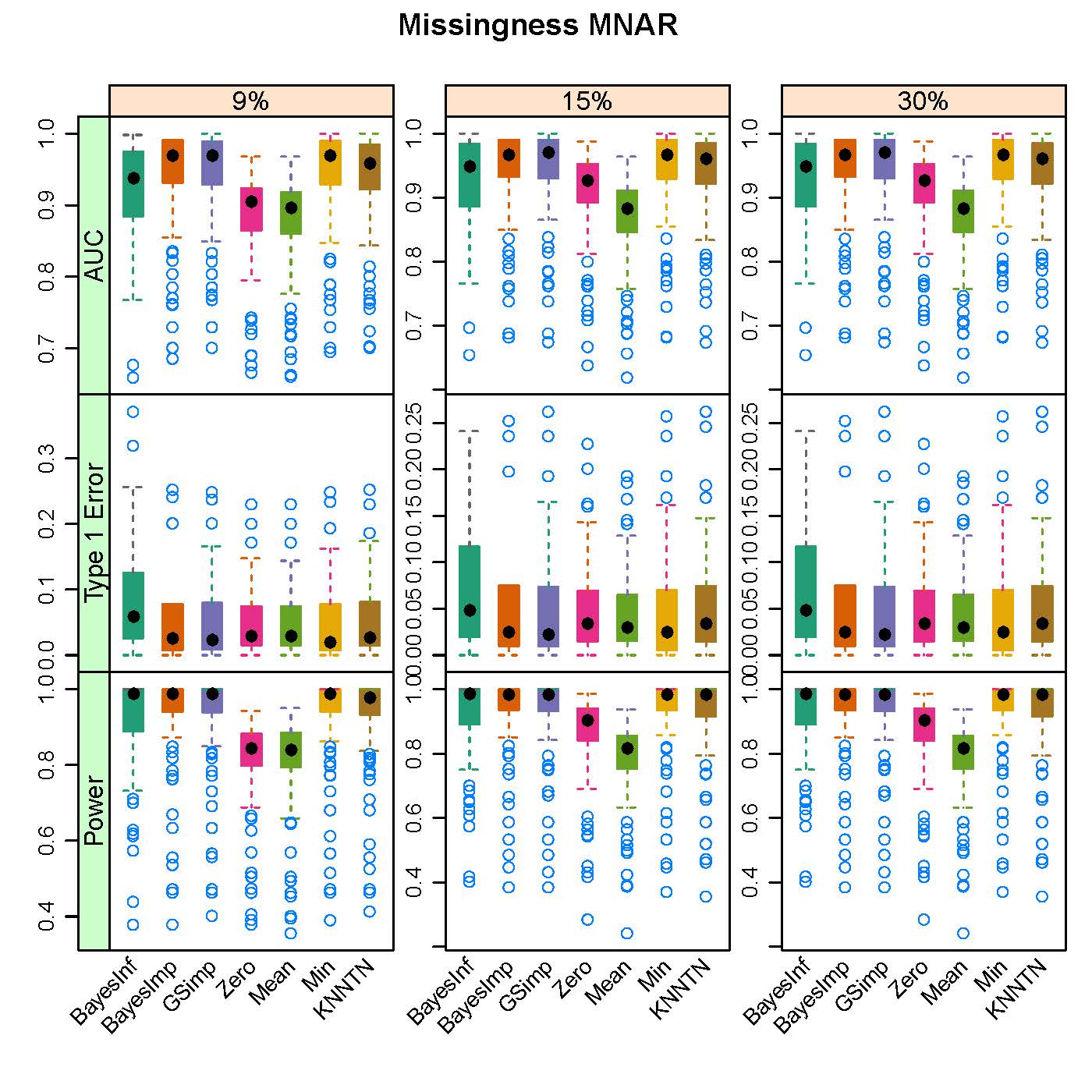
**

**Figure S10**: Box plots for Power, Type 1 Error and AUC for Bayesian, GSimp, Zero, Min, Mean and KNNTN methods for 100 datasets, 50 samples by 400 metabolites. Total missing was considered at 9%, 15%, and 30% and completely MNAR.


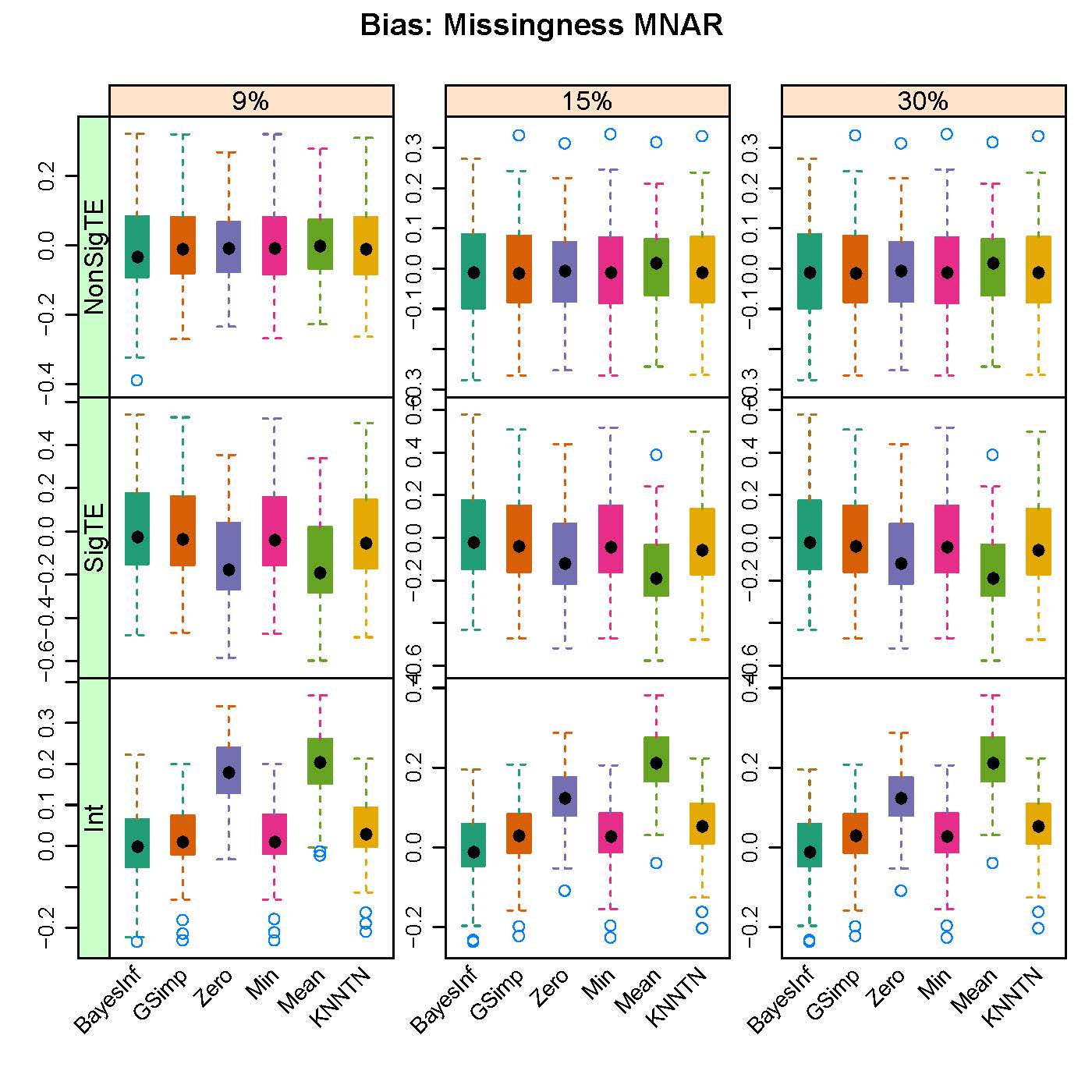


**Figure S11**: Box plots for Bias for Bayesian, GSimp, Zero, Min, Mean and KNNTN methods for 100 datasets, 50 samples by 400 metabolites. Total missing was considered at 9%, 15%, and 30% and completely MNAR.


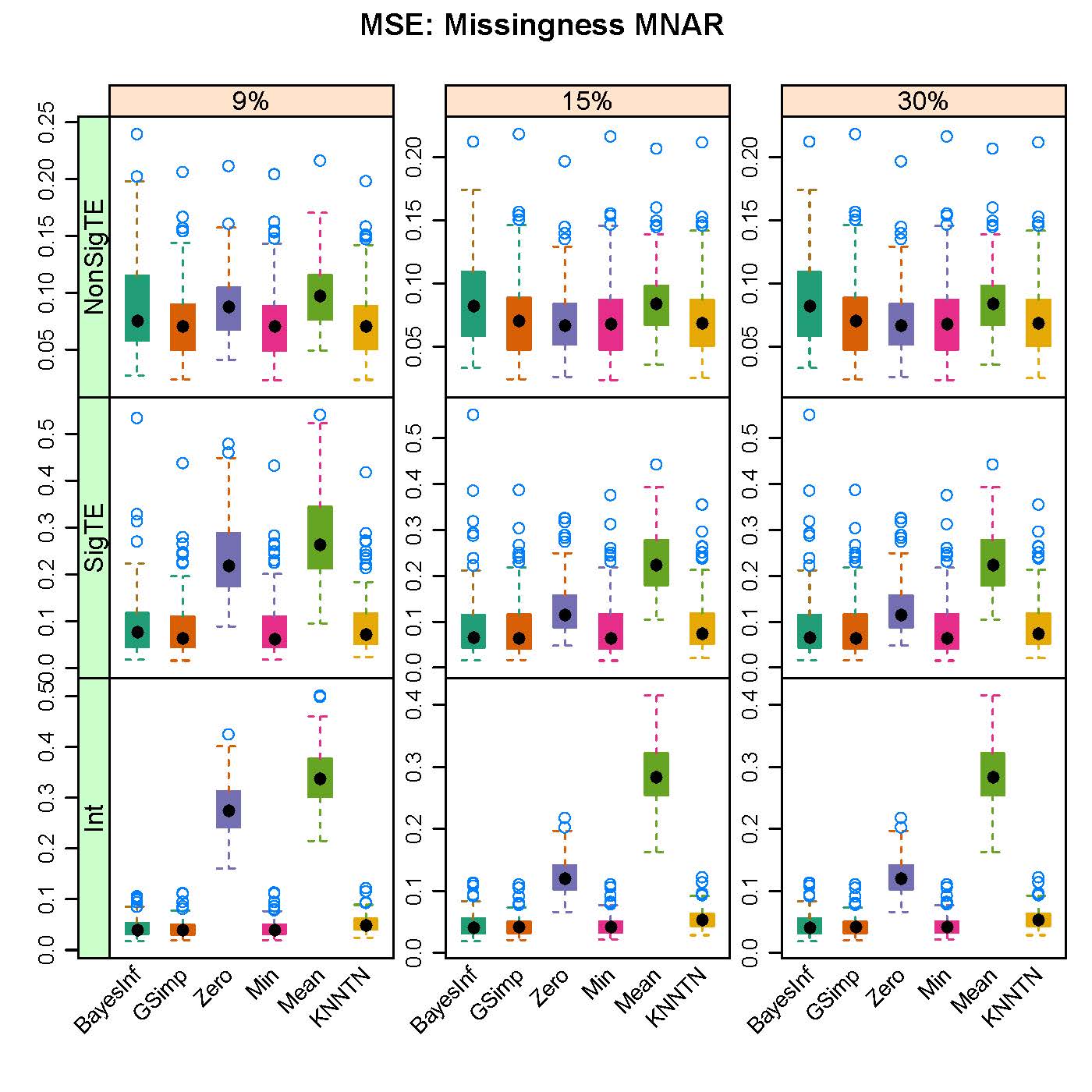


**Figure S12**: Box plots for MSE for Bayesian, GSimp, Zero, Min, Mean and KNNTN methods for 100 datasets, 50 samples by 400 metabolites. Total missing was considered at 9%, 15%, and 30% and completely MNAR.


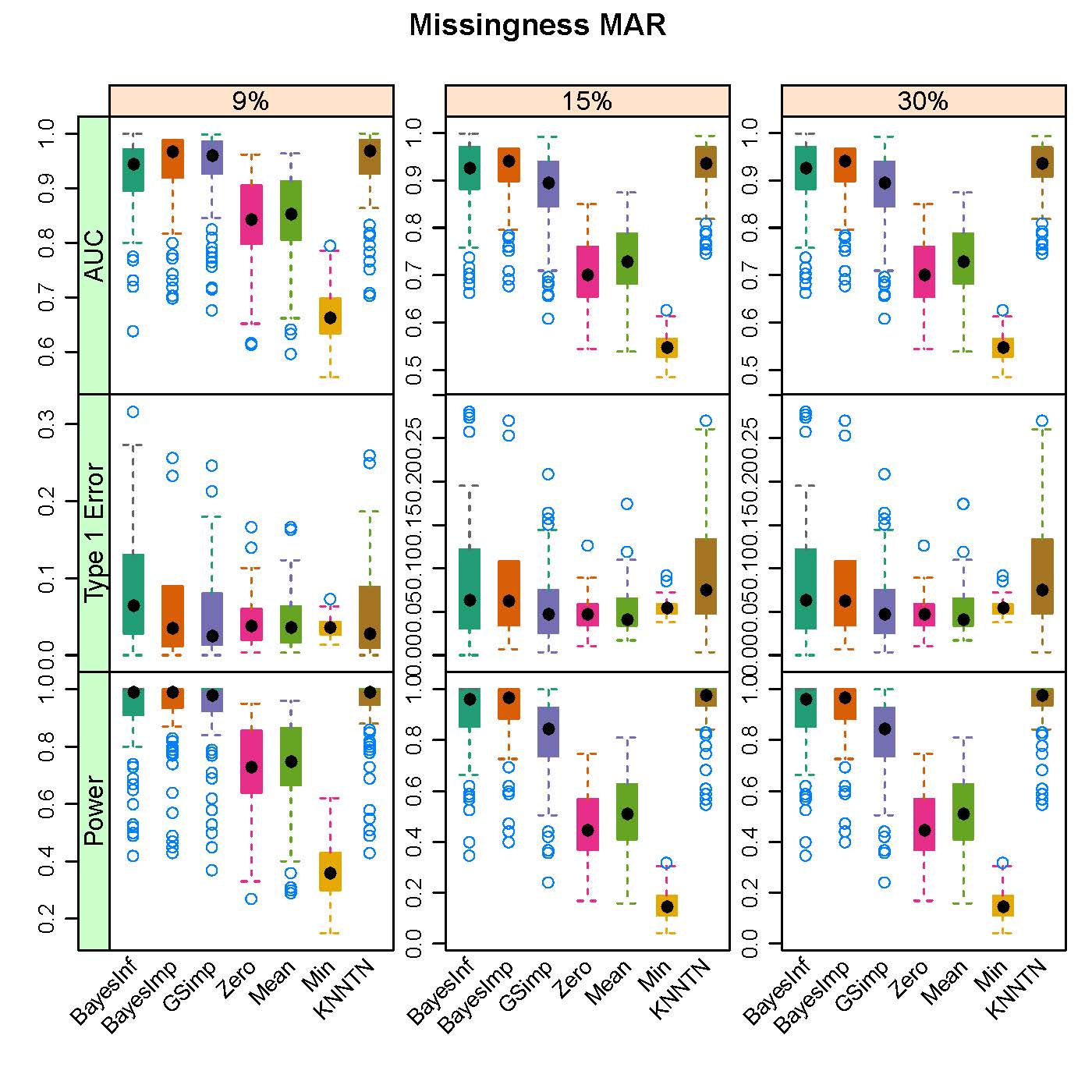


**Figure S13**: Box plots for Power, Type 1 Error and AUC for Bayesian, GSimp, Zero, Min, Mean and KNNTN methods for 100 datasets, 50 samples by 400 metabolites. Total missing was considered at 9%, 15%, and 30% and completely MAR.


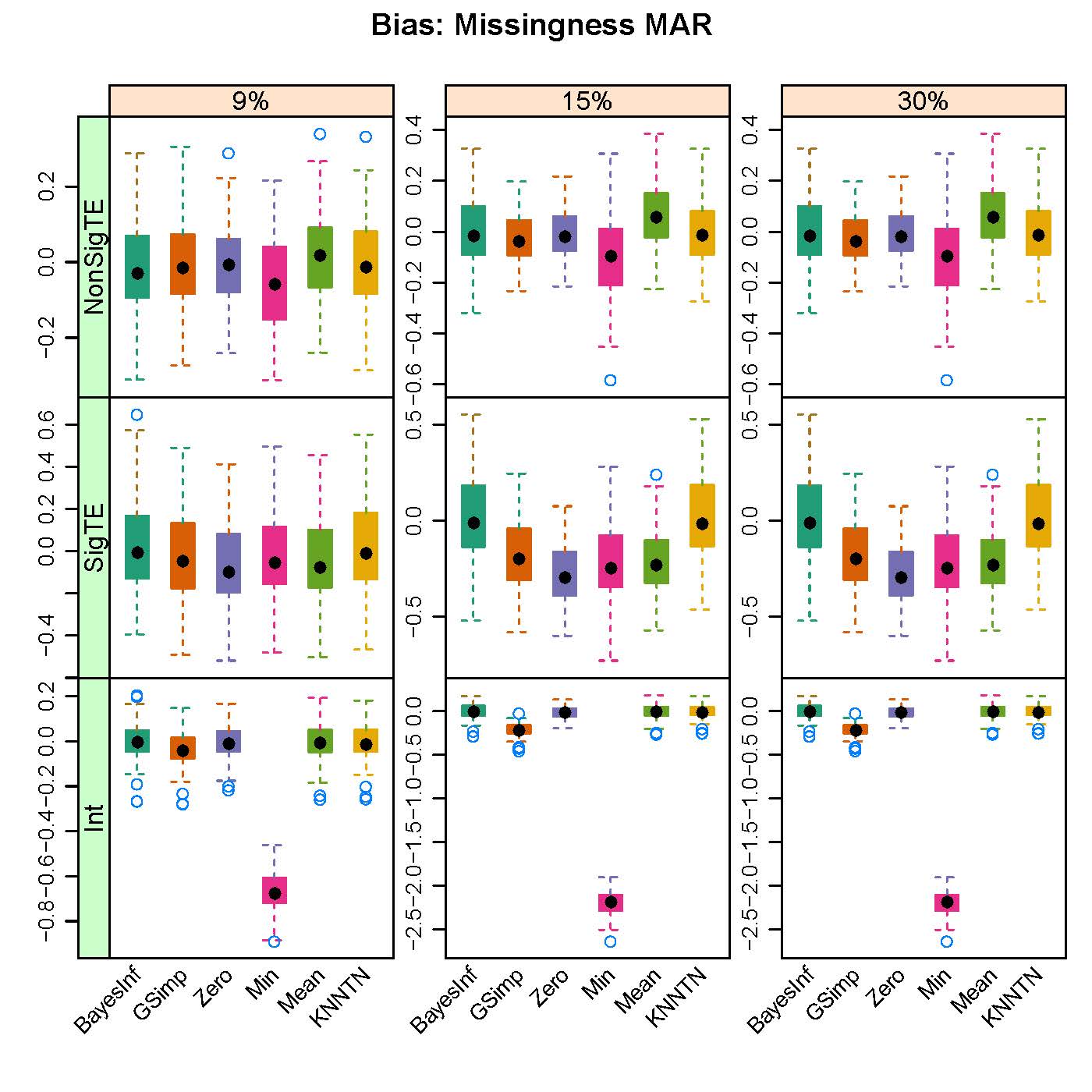


**Figure S14**: Box plots for Bias for Bayesian, GSimp, Zero, Min, Mean and KNNTN methods for 100 datasets, 50 samples by 400 metabolites. Total missing was considered at 9%, 15%, and 30% and completely MAR.


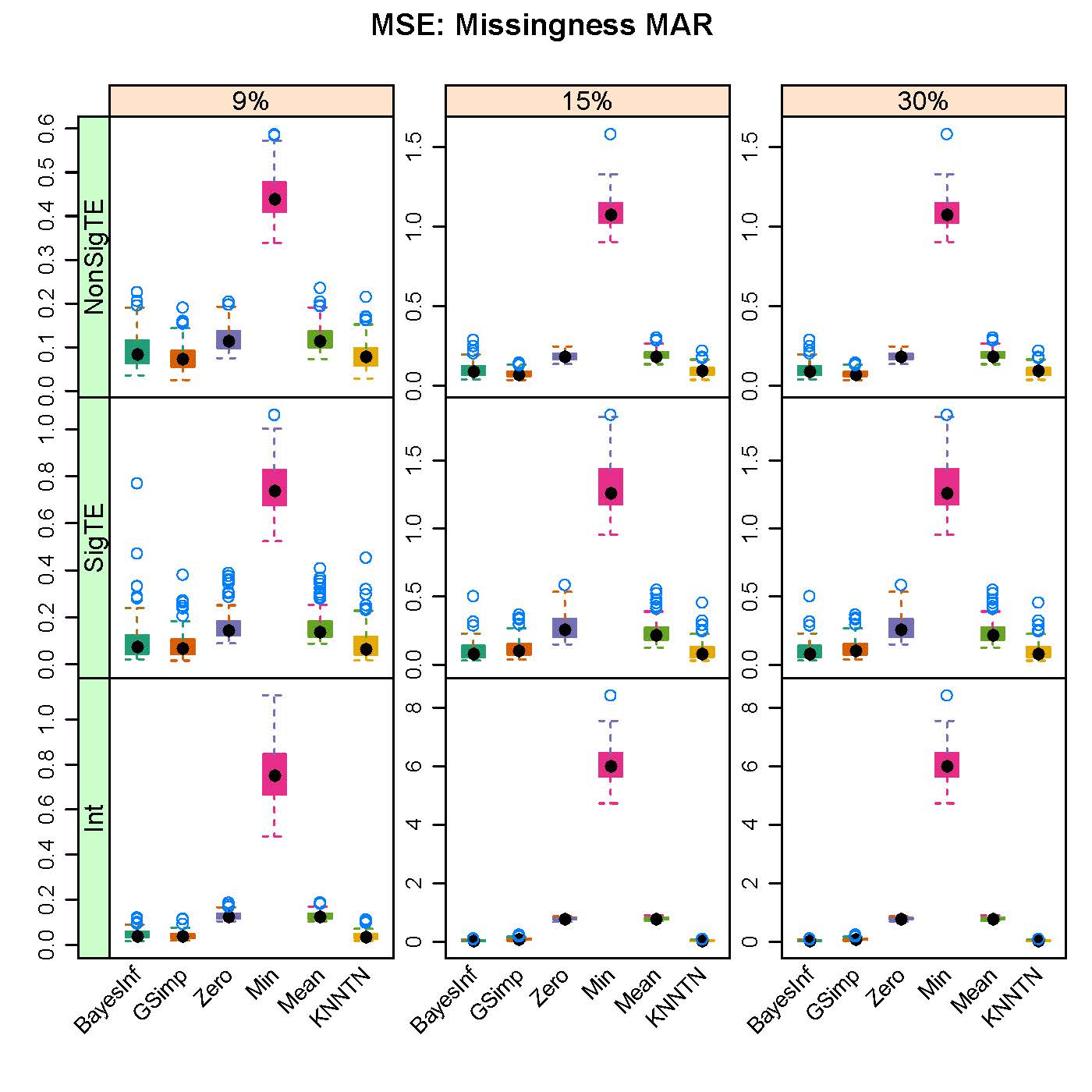


**Figure S15**: Box plots for MSE for Bayesian, GSimp, Zero, Min, Mean and KNNTN methods for 100 datasets, 50 samples by 400 metabolites. Total missing was considered at 9%, 15%, and 30% and completely MAR.


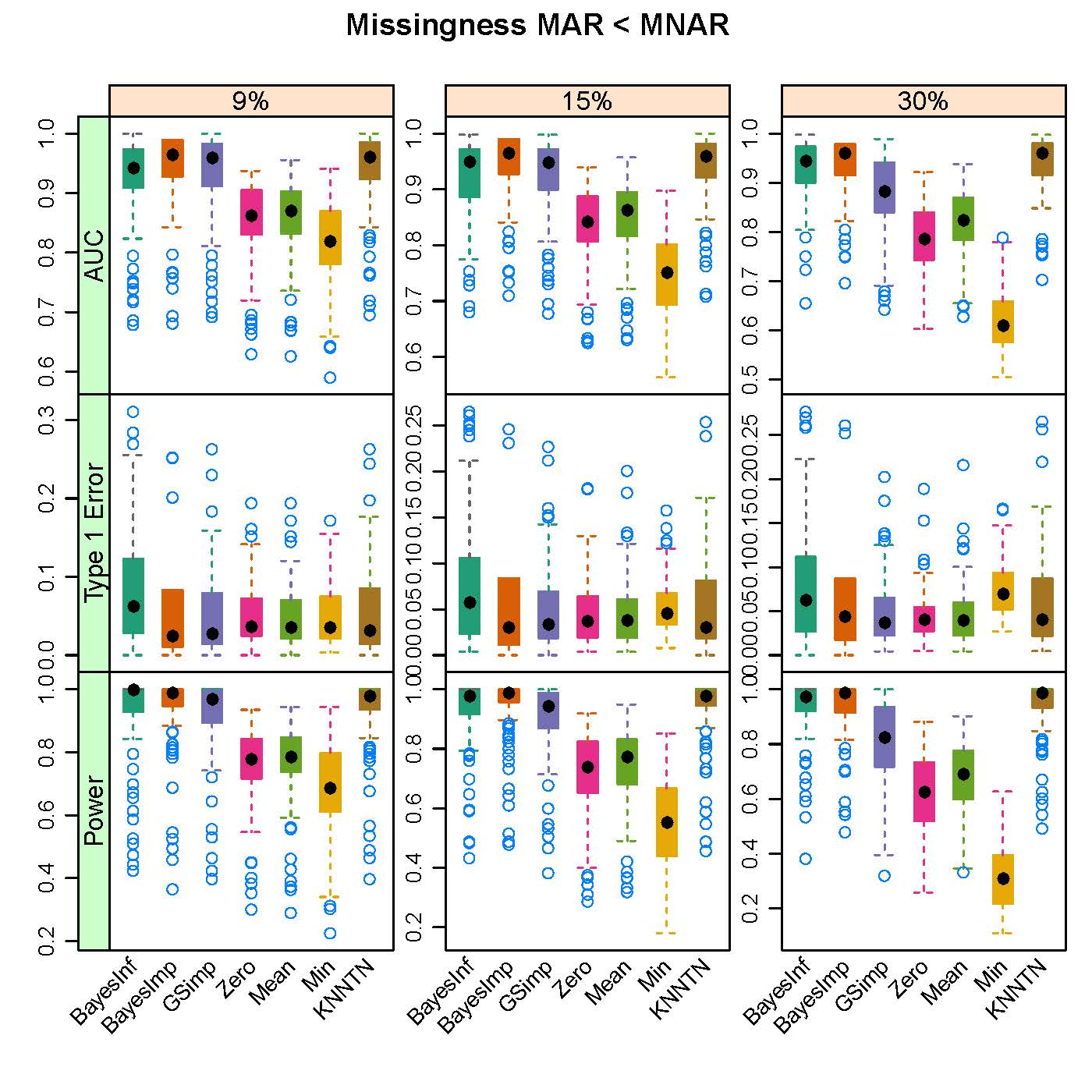


**Figure S16**: Box plots for Power, Type 1 Error and AUC for Bayesian, GSimp, Zero, Min, Mean and KNNTN methods for 100 datasets, 50 samples by 400 metabolites. Total missing was considered at 9%, 15%, and 30% and within each missing MNAR is greater than MAR.


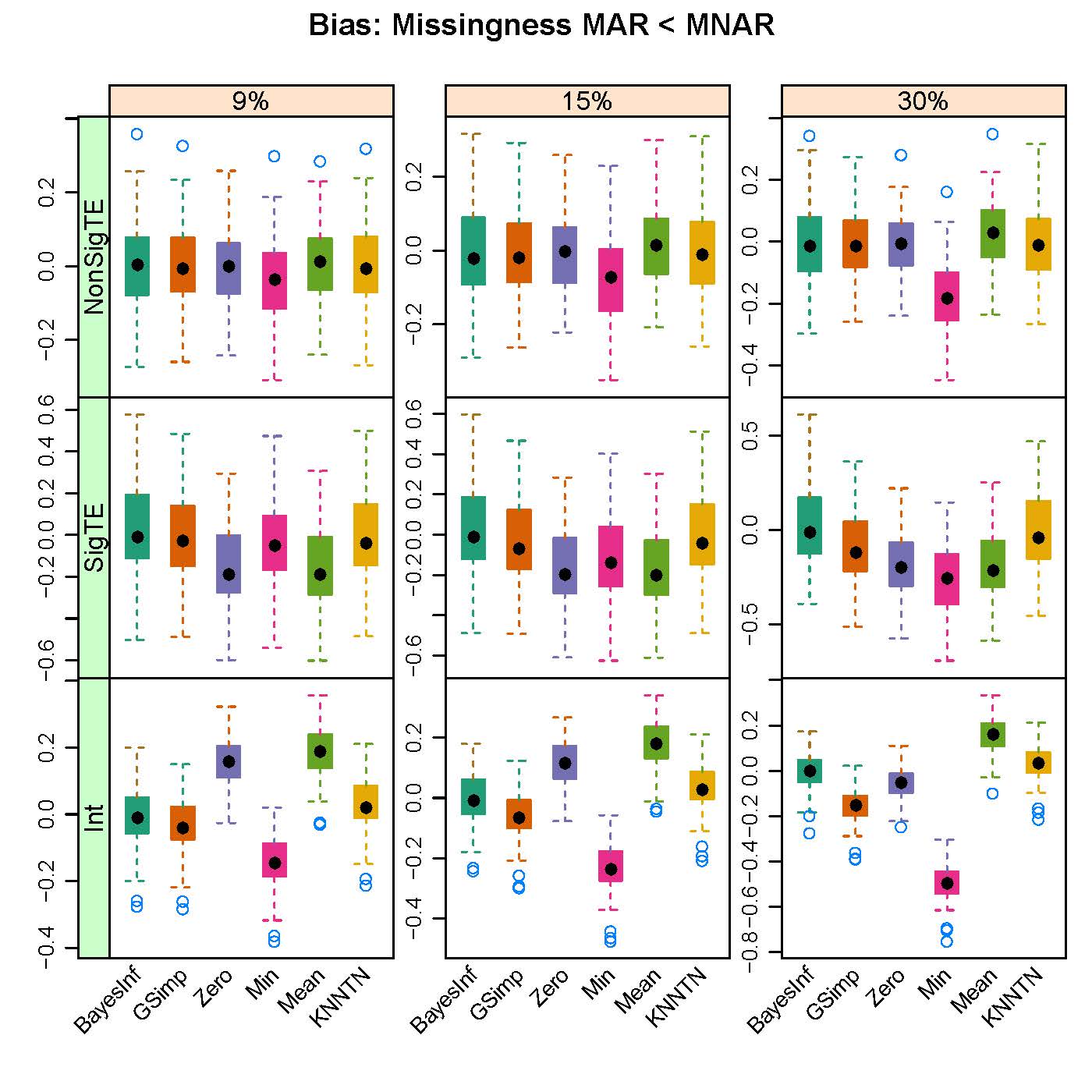


**Figure S17**: Box plots for Bias for Bayesian, GSimp, Zero, Min, Mean and KNNTN methods for 100 datasets, 50 samples by 400 metabolites. Total missing was considered at 9%, 15%, and 30% and within each missing MNAR is greater than MAR.


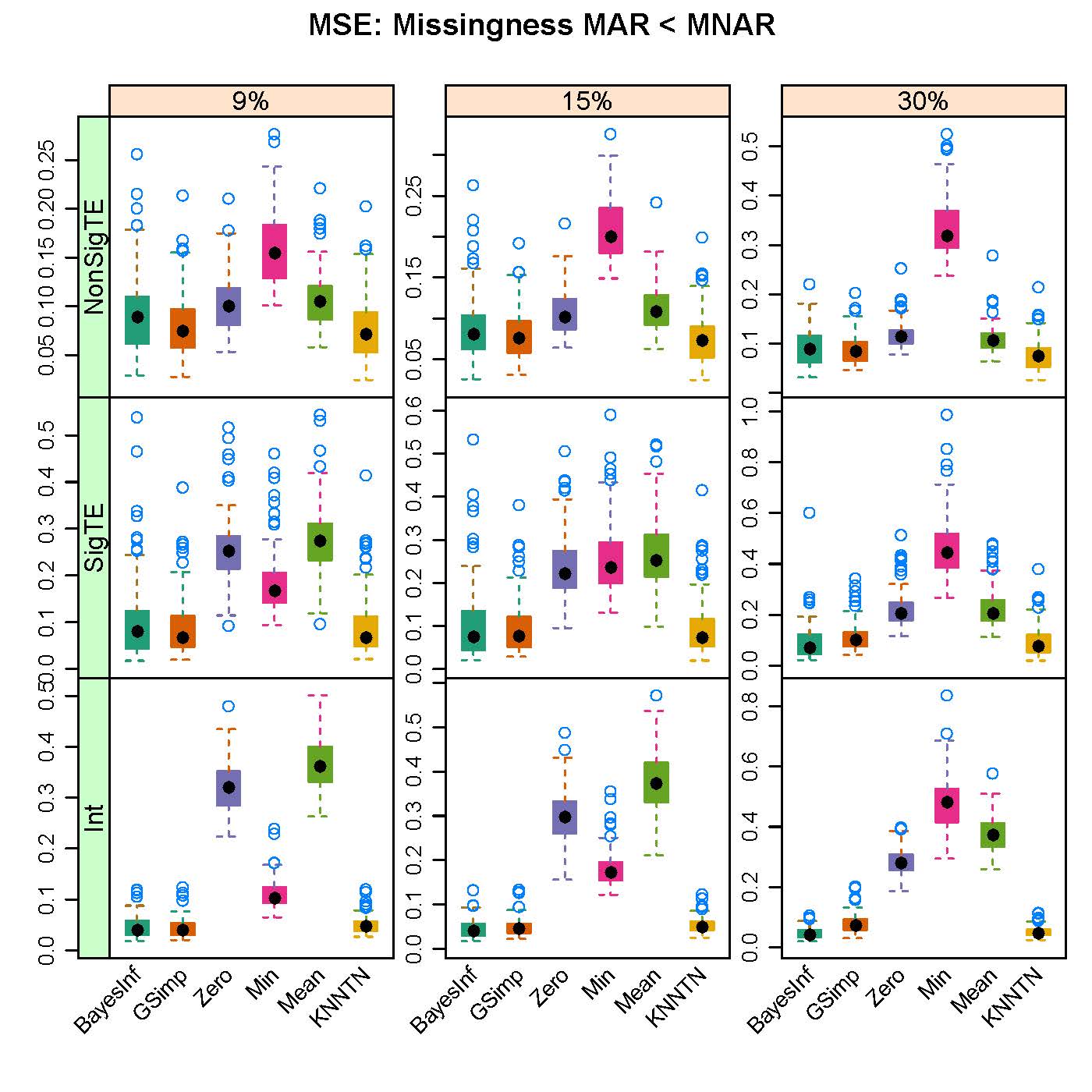


**Figure S18**: Box plots for MSE for Bayesian, GSimp, Zero, Min, Mean and KNNTN methods for 100 datasets, 50 samples by 400 metabolites. Total missing was considered at 9%, 15%, and 30% and within each missing MNAR is greater than MAR.

**
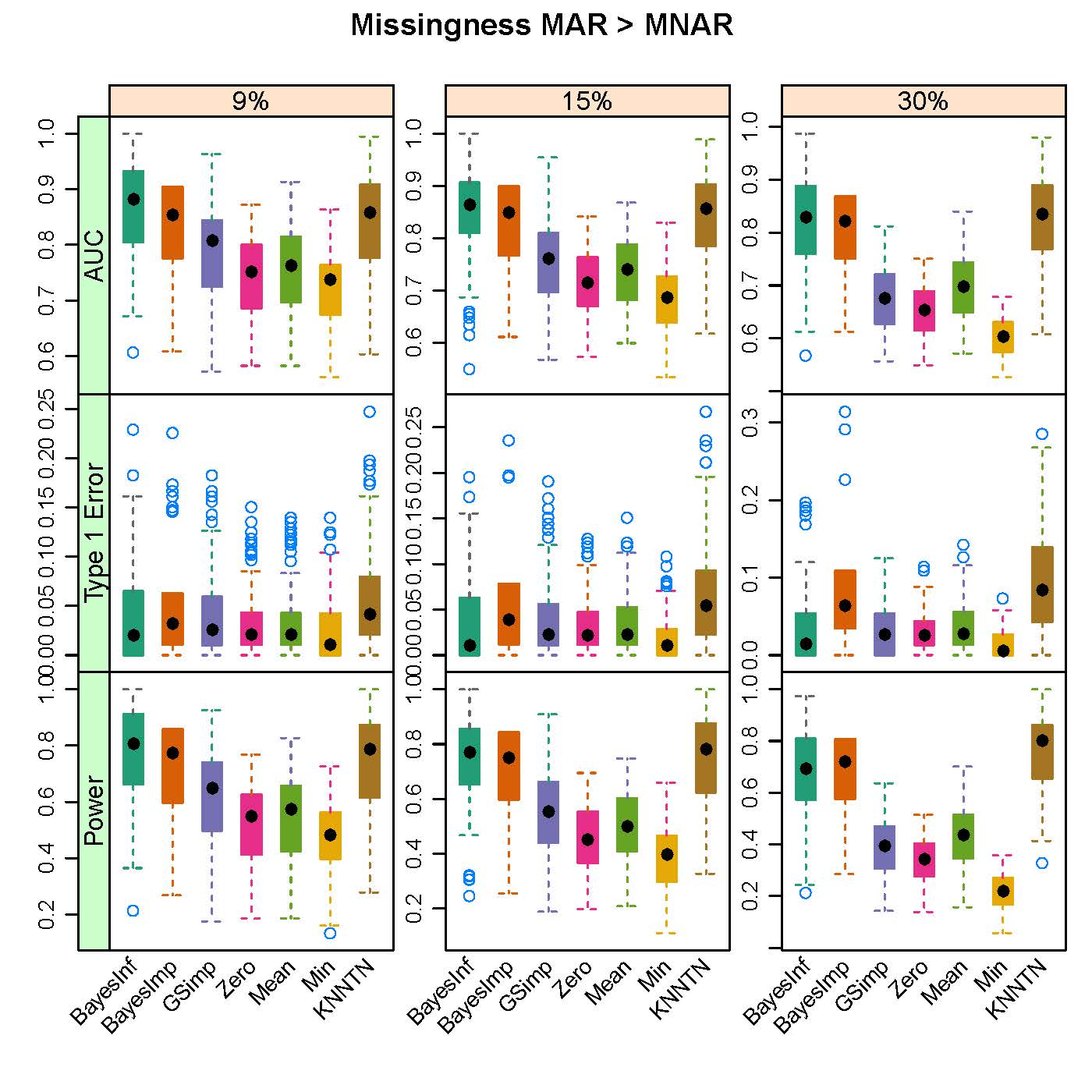
**

**Figure S19**: Box plots for Power, Type 1 Error and AUC for Bayesian, GSimp, Zero, Min, Mean and KNNTN methods for 100 datasets, 10 samples by 200 metabolites (effect size = 1.6). Total missing was considered at 9%, 15%, and 30% and within each missing MNAR is less than MAR.


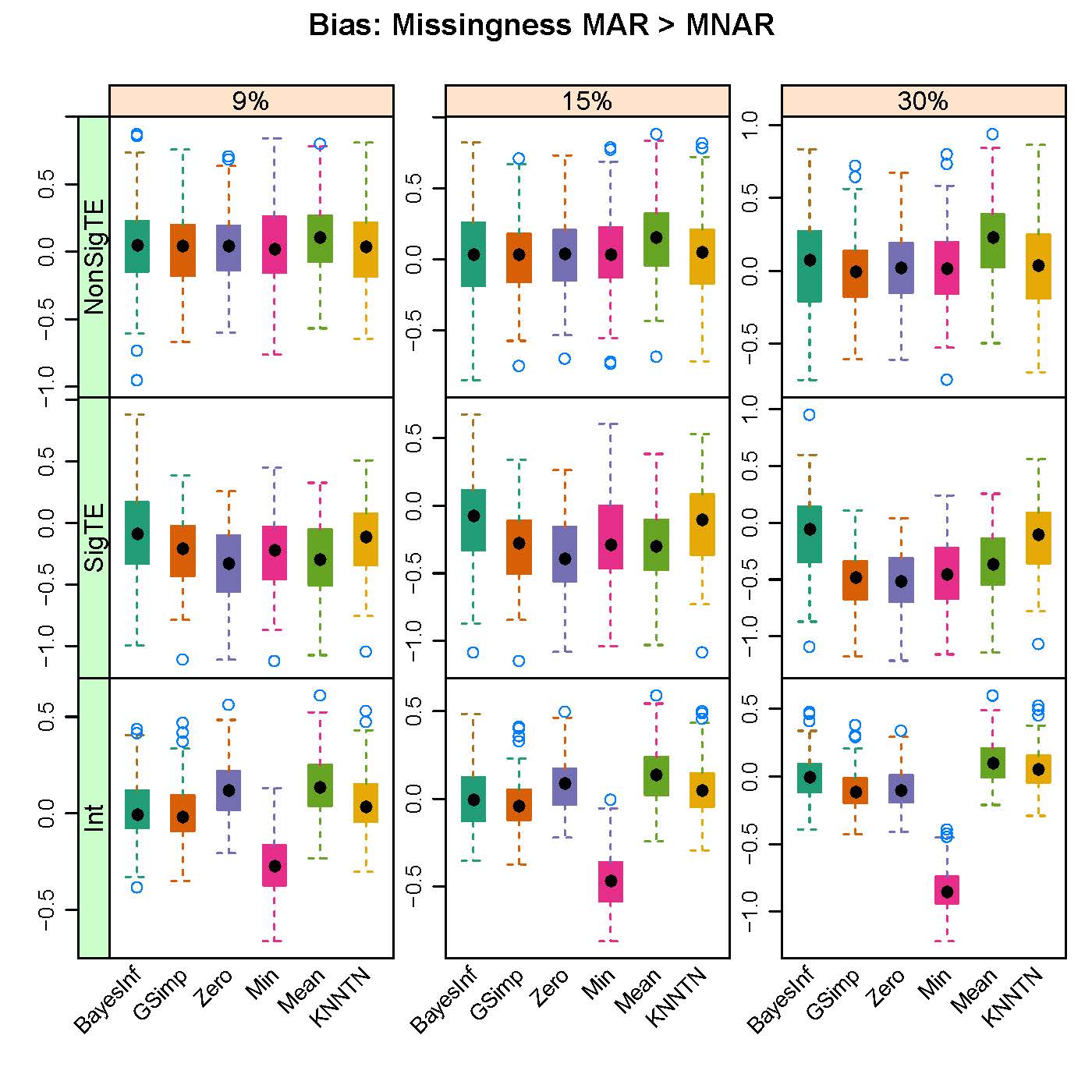


**Figure S20**: Box plots for Bias for Bayesian, GSimp, Zero, Min, Mean and KNNTN methods for 100 datasets, 10 samples by 200 metabolites (effect size = 1.6). Total missing was considered at 9%, 15%, and 30% and within each missing MNAR is less than MAR.


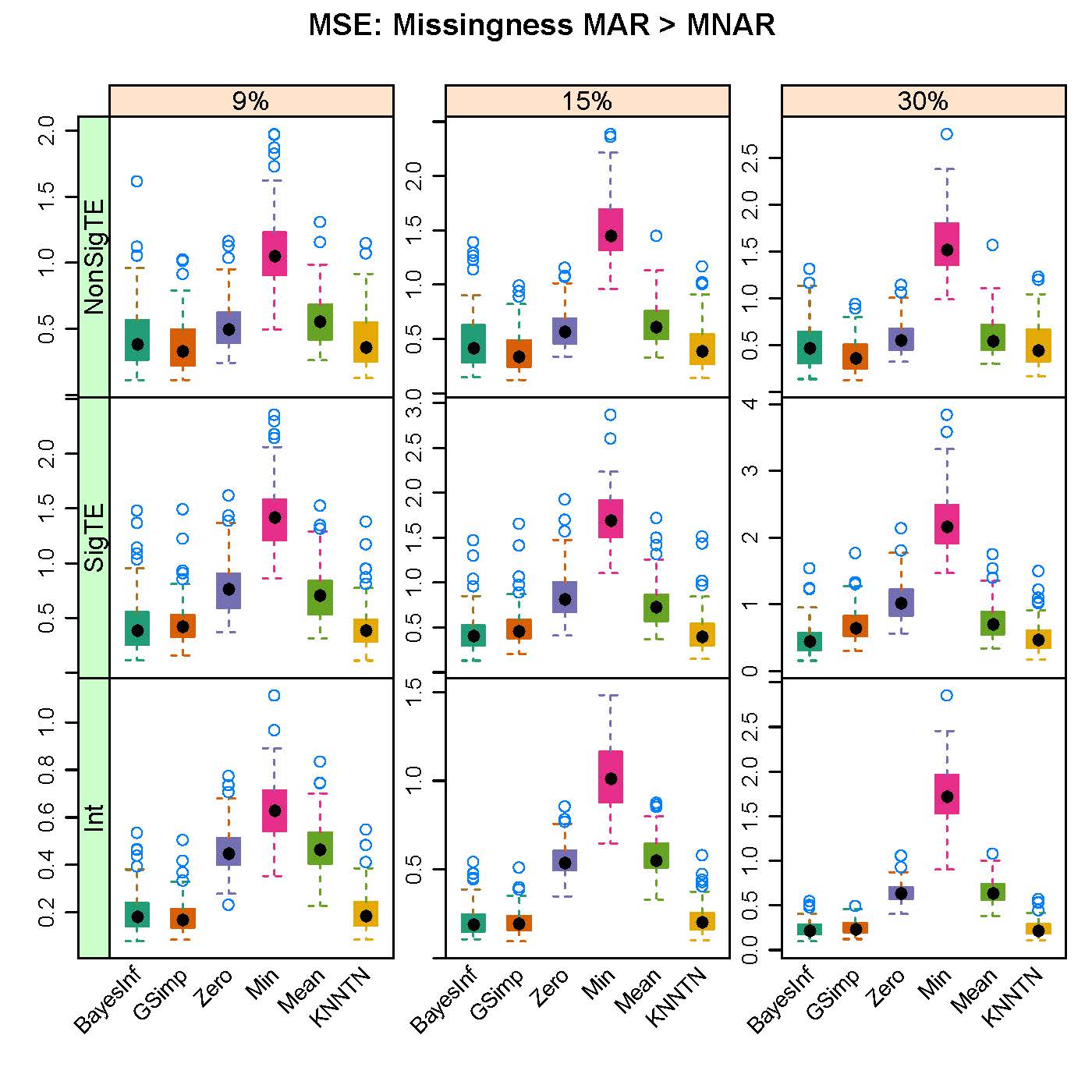


**Figure S21**: Box plots for MSE for Bayesian, GSimp, Zero, Min, Mean and KNNTN methods for 100 datasets, 10 samples by 200 metabolites (effect size = 1.6). Total missing was considered at 9%, 15%, and 30% and within each missing MNAR is less than MAR.


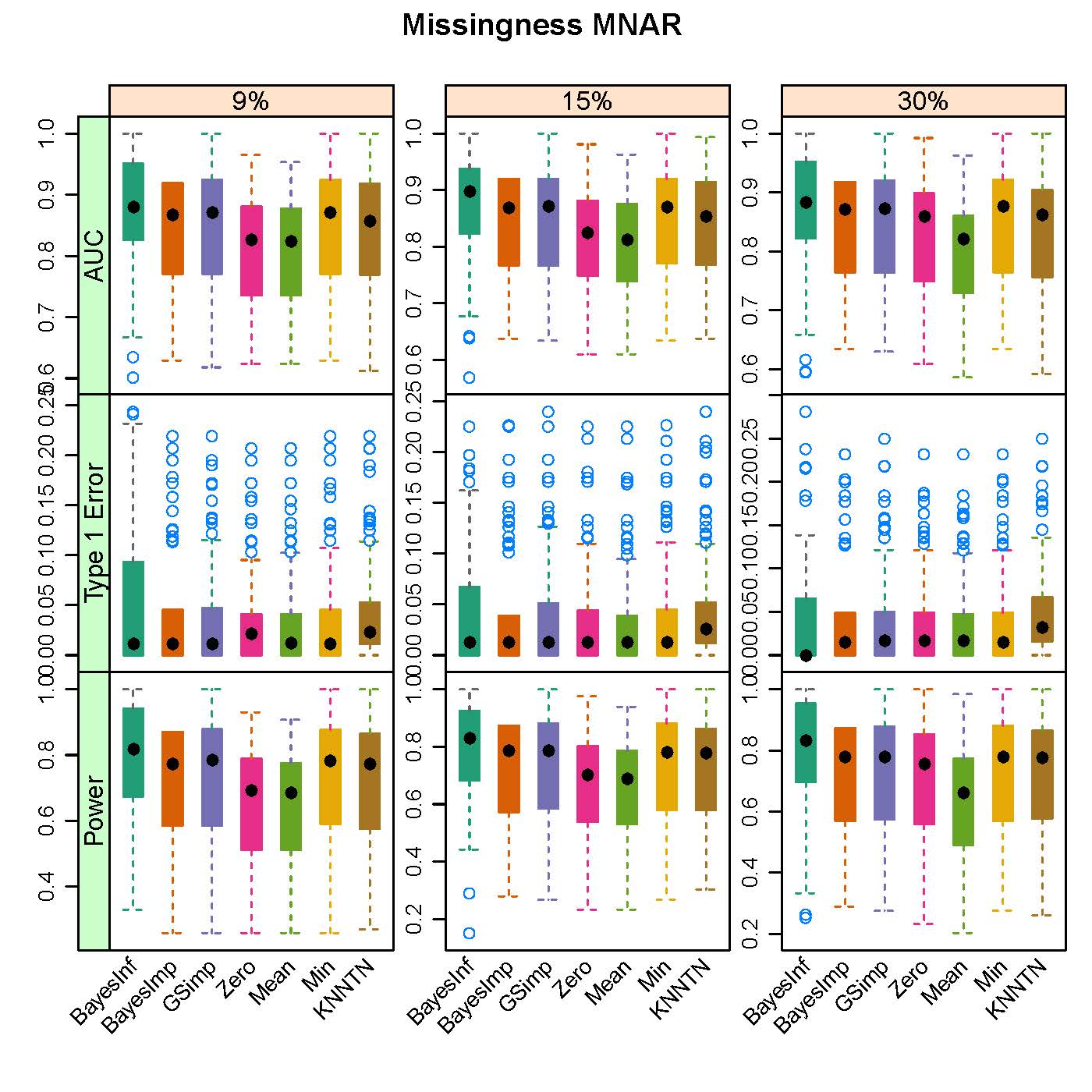


**Figure S22**: Box plots for Power, Type 1 Error and AUC for Bayesian, GSimp, Zero, Min, Mean and KNNTN methods for 100 datasets, 10 samples by 200 metabolites (effect size = 1.6). Total missing was considered at 9%, 15%, and 30% and completely MNAR.


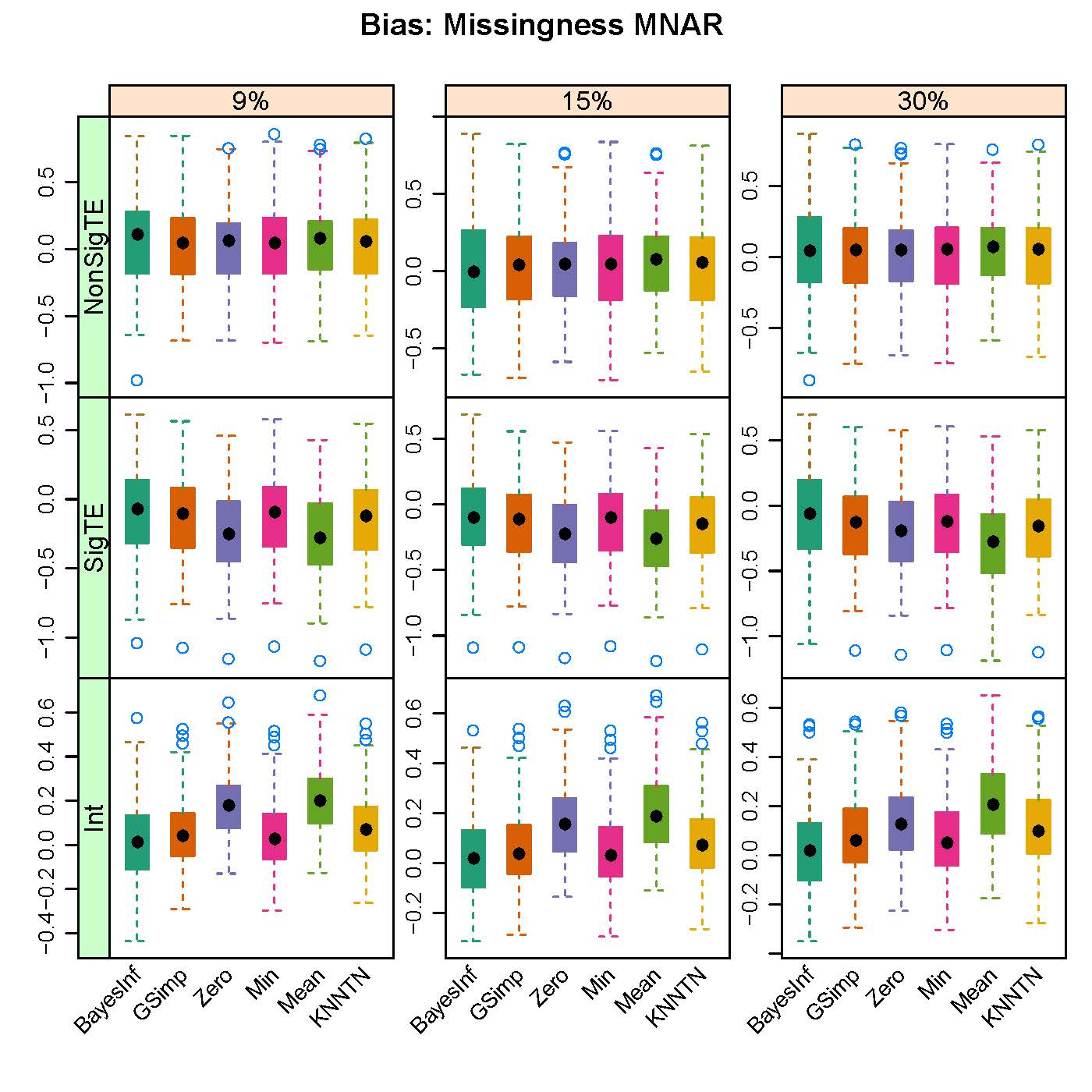


**Figure S23**: Box plots for Bias for Bayesian, GSimp, Zero, Min, Mean and KNNTN methods for 100 datasets, 10 samples by 200 metabolites (effect size = 1.6). Total missing was considered at 9%, 15%, and 30% and completely MNAR.


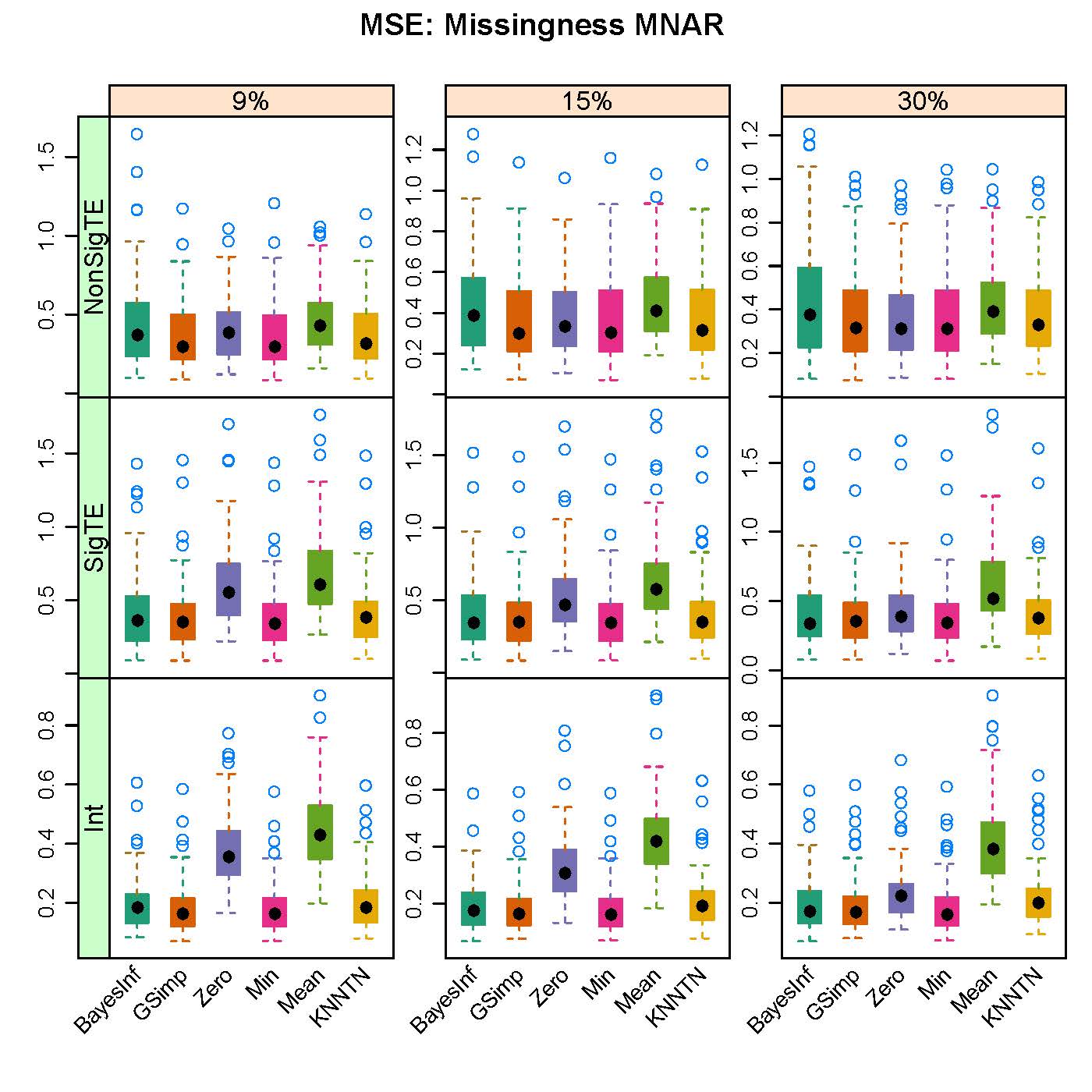


**Figure S24**: Box plots for MSE for Bayesian, GSimp, Zero, Min, Mean and KNNTN methods for 100 datasets, 10 samples by 200 metabolites (effect size = 1.6). Total missing was considered at 9%, 15%, and 30% and completely MNAR.


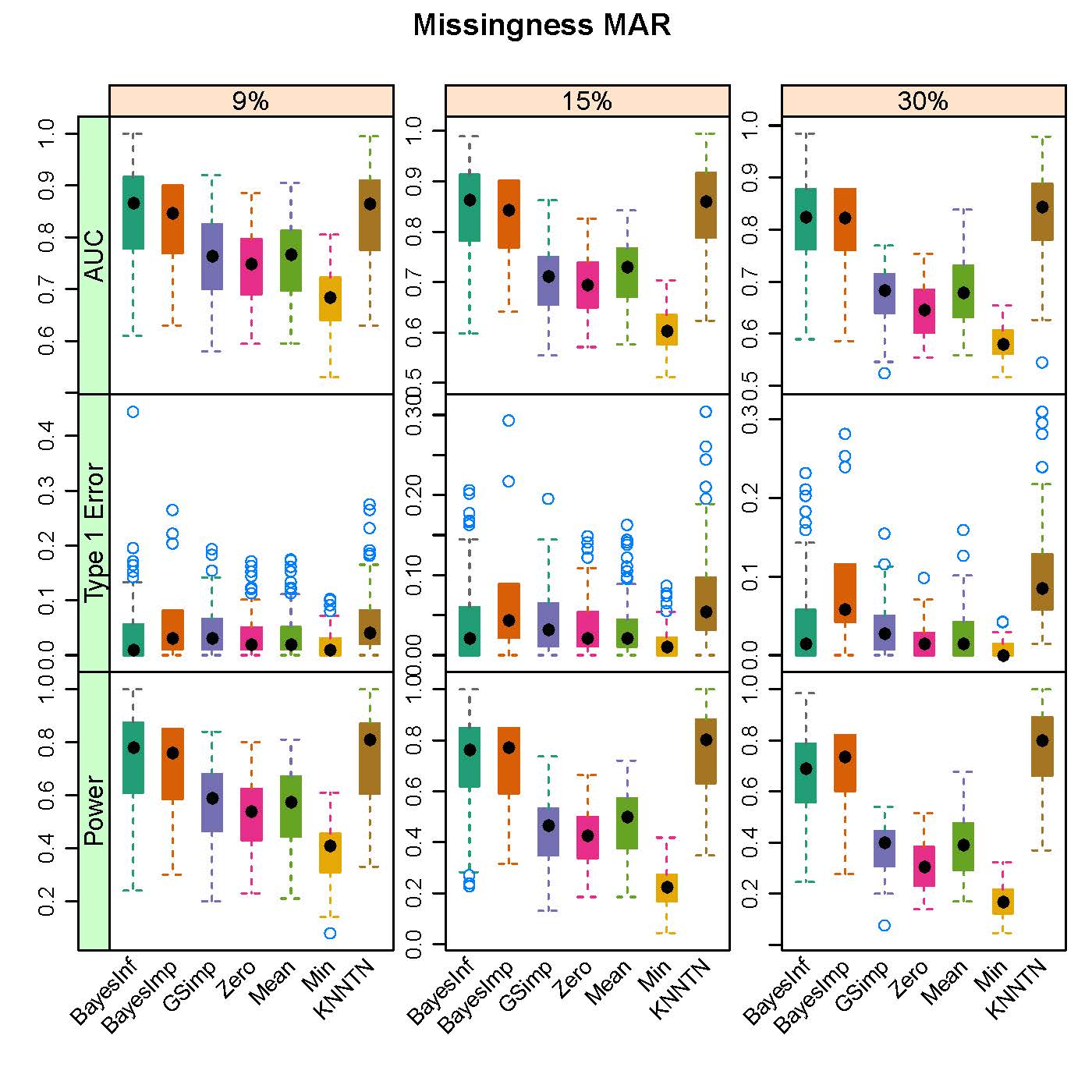


**Figure S25**: Box plots for Power, Type 1 Error and AUC for Bayesian, GSimp, Zero, Min, Mean and KNNTN methods for 100 datasets, 10 samples by 200 metabolites (effect size = 1.6). Total missing was considered at 9%, 15%, and 30% and completely MAR.


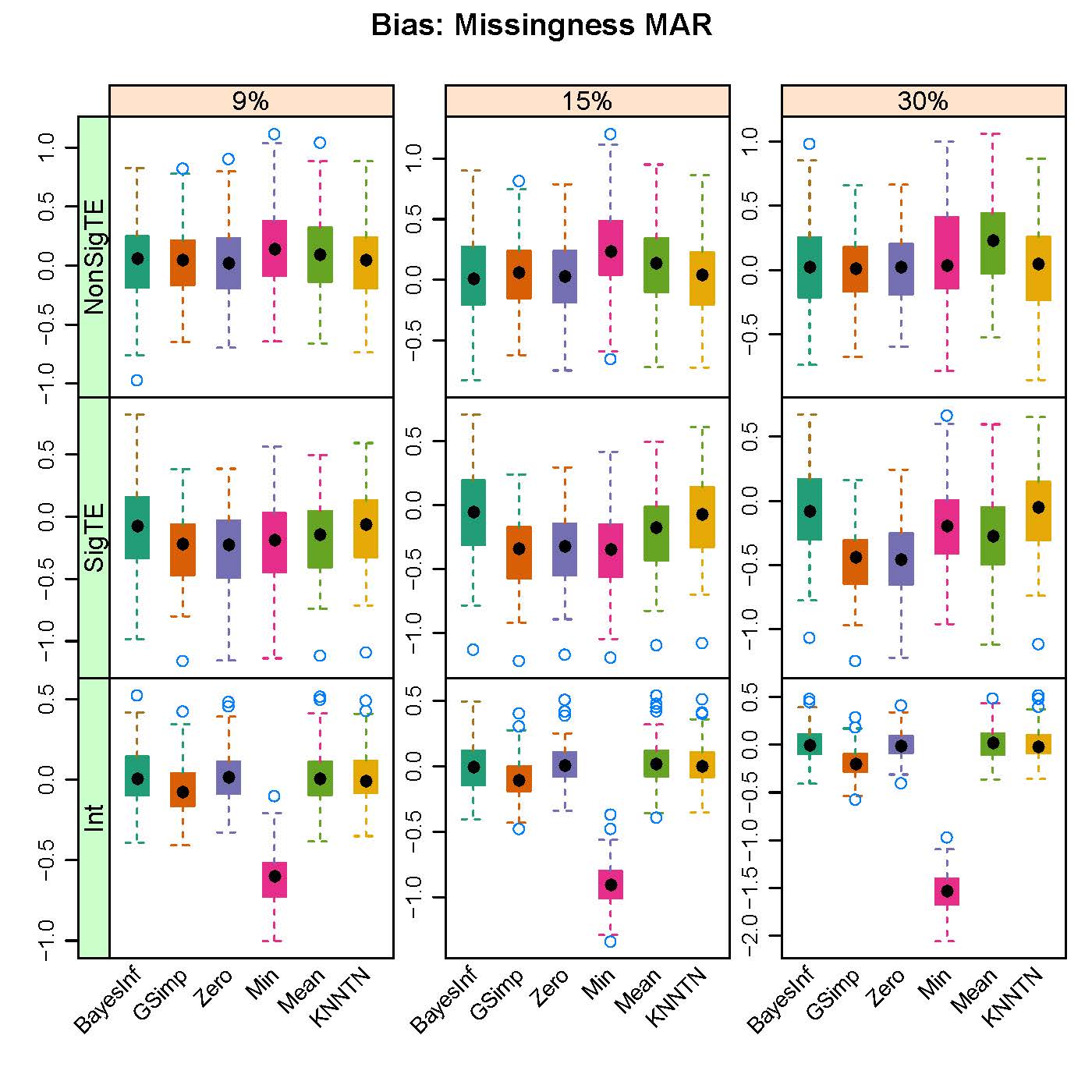


**Figure S26:** Box plots for Bias for Bayesian, GSimp, Zero, Min, Mean and KNNTN methods for 100 datasets, 10 samples by 200 metabolites (effect size = 1.6). Total missing was considered at 9%, 15%, and 30% and completely MAR.


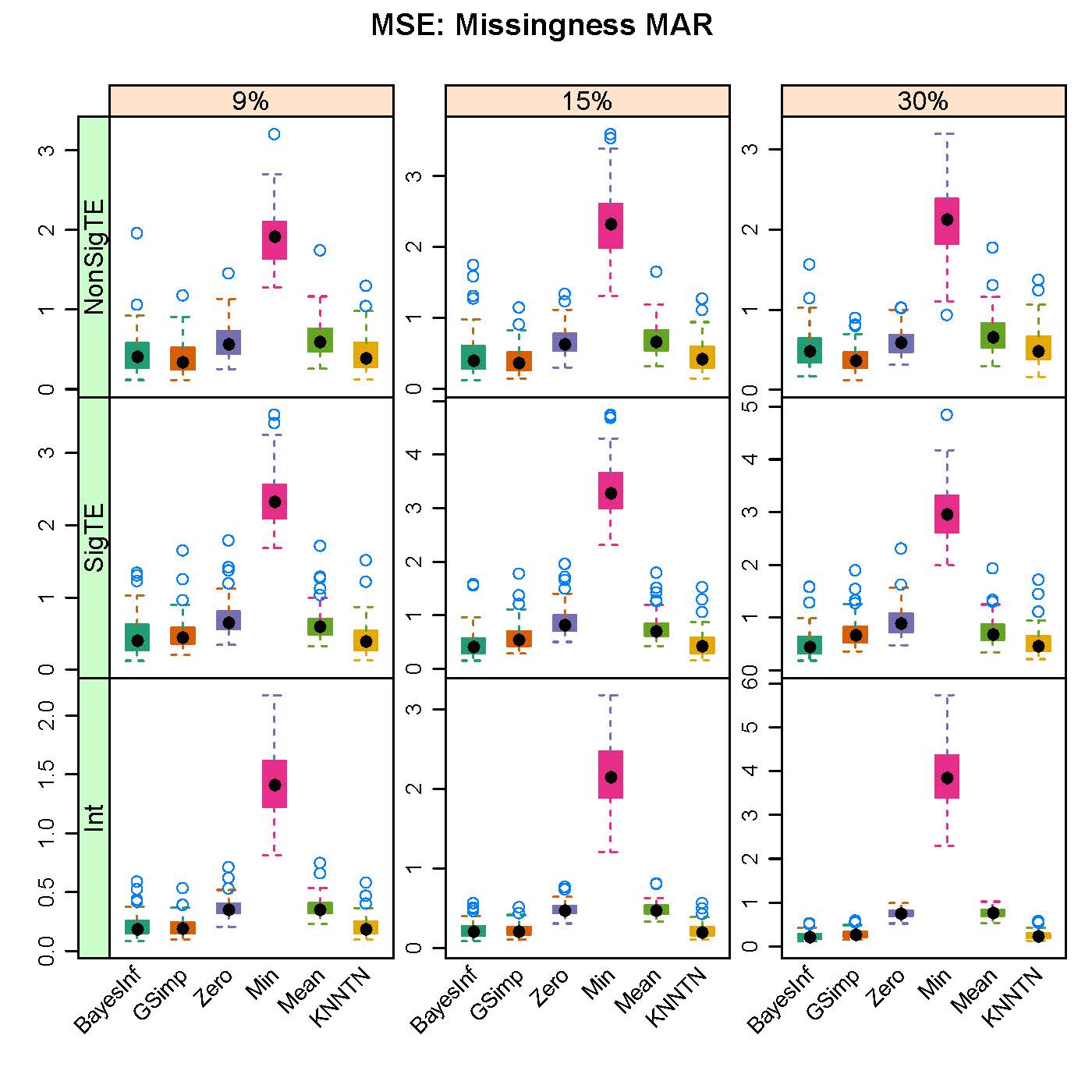


**Figure S27**: Box plots for MSE for Bayesian, GSimp, Zero, Min, Mean and KNNTN methods for 100 datasets, 10 samples by 200 metabolites (effect size = 1.6). Total missing was considered at 9%, 15%, and 30% and completely MAR.


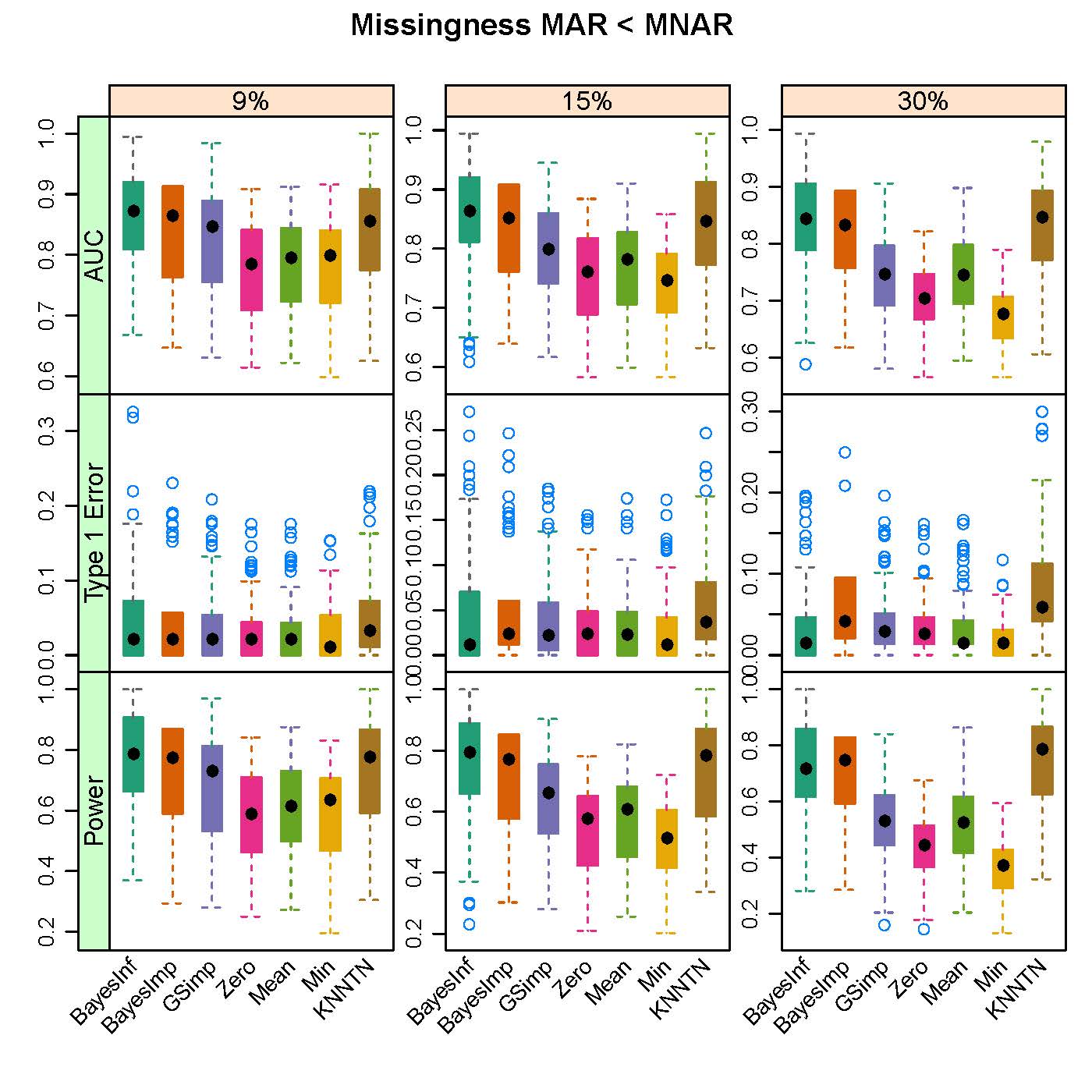


**Figure S28**: Box plots for Power, Type 1 Error and AUC for Bayesian, GSimp, Zero, Min, Mean and KNNTN methods for 100 datasets, 10 samples by 200 metabolites (effect size = 1.6). Total missing was considered at 9%, 15%, and 30% and within each missing MNAR is greater than MAR.


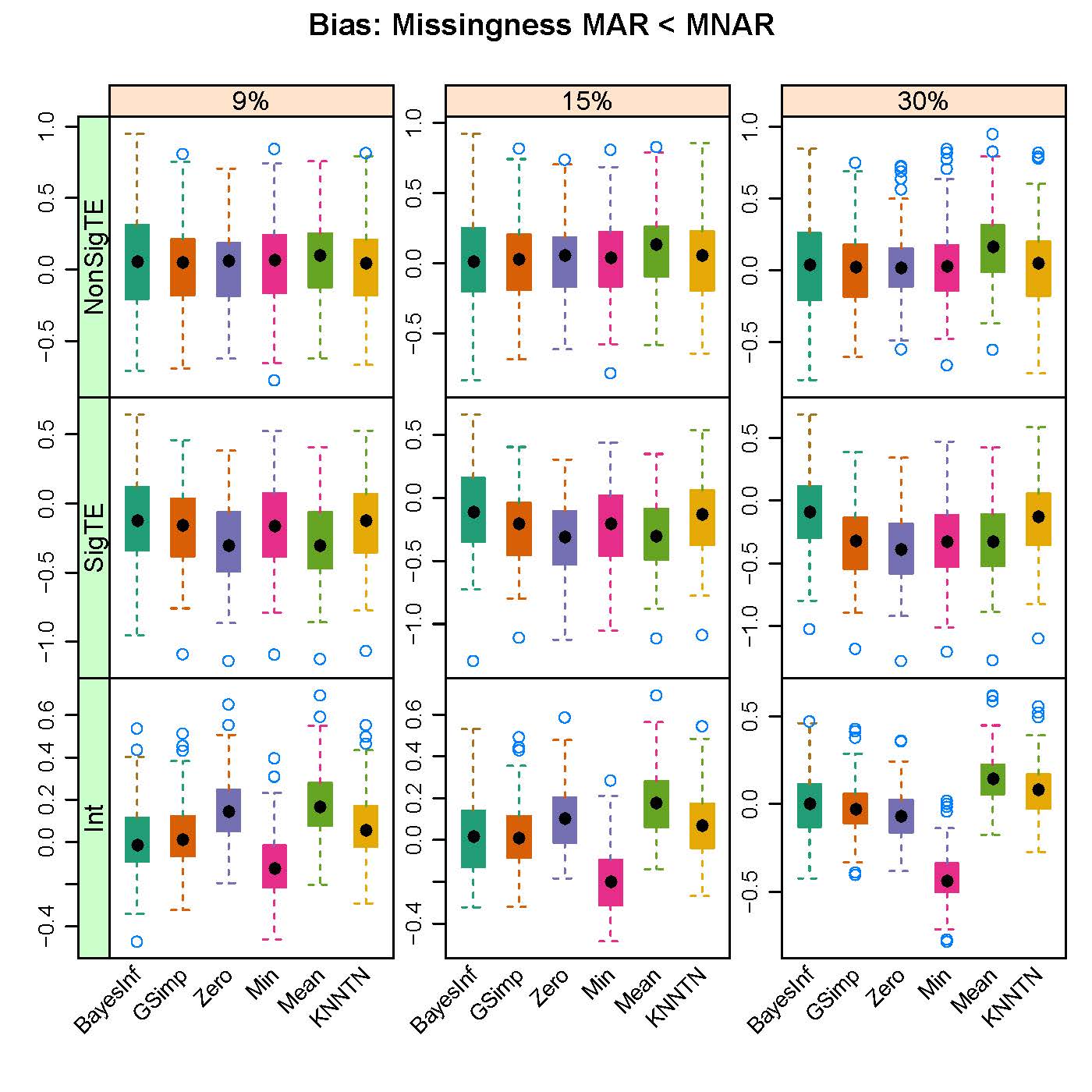


**Figure S29**: Box plots for Bias for Bayesian, GSimp, Zero, Min, Mean and KNNTN methods for 100 datasets, 10 samples by 200 metabolites (effect size = 1.6). Total missing was considered at 9%, 15%, and 30% and within each missing MNAR is greater than MAR.


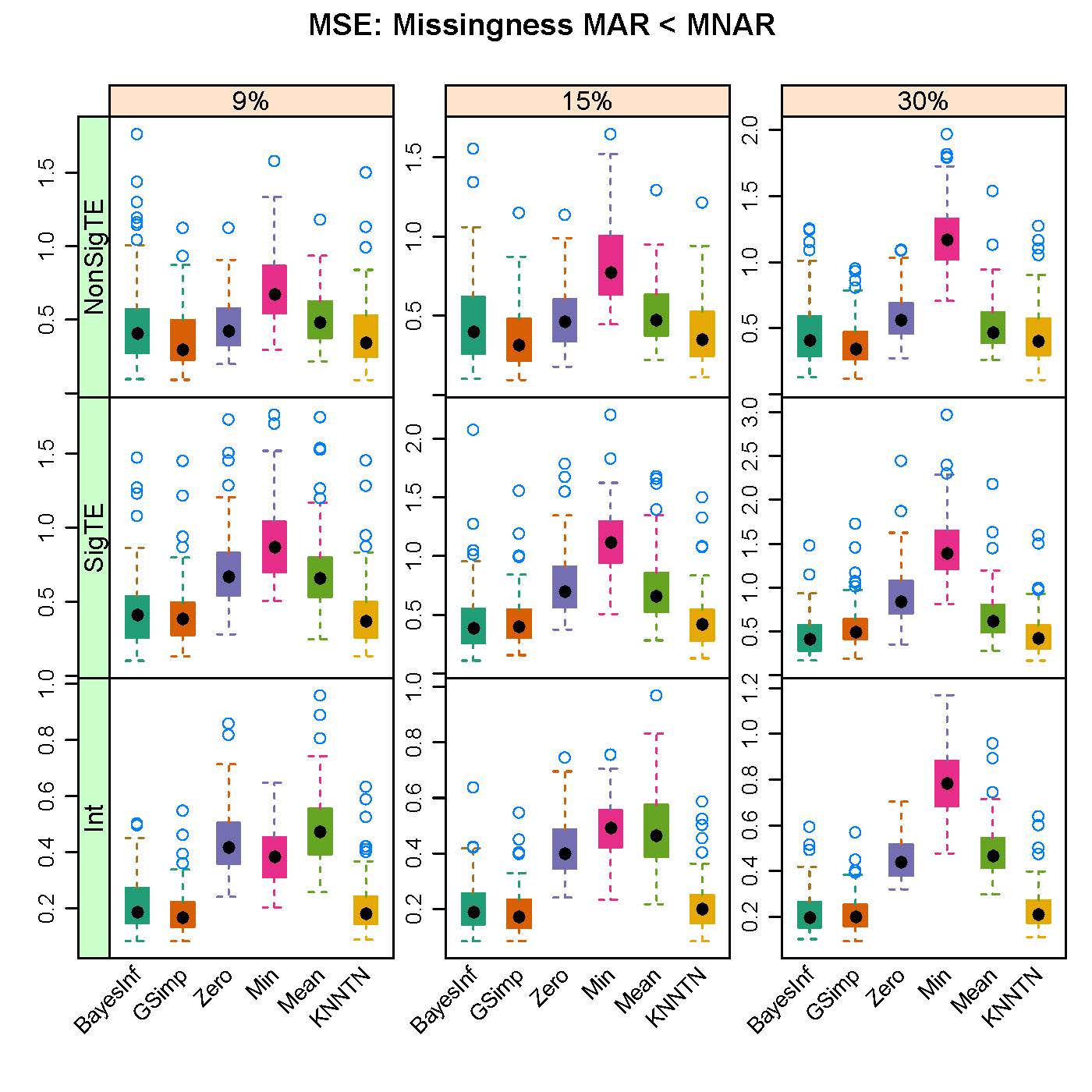


**Figure S30**: Box plots for MSE for Bayesian, GSimp, Zero, Min, Mean and KNNTN methods for 100 datasets, 10 samples by 200 metabolites (effect size = 1.6). Total missing was considered at 9%, 15%, and 30% and within each missing MNAR is greater than MAR.
